# Transcriptional Activity, Chromosomal Distribution and Expression Effects of Transposable Elements in *Coffea* Genomes

**DOI:** 10.1371/journal.pone.0078931

**Published:** 2013-11-11

**Authors:** Fabrício R. Lopes, Daudi Jjingo, Carlos R. M. da Silva, Alan C. Andrade, Pierre Marraccini, João B. Teixeira, Marcelo F. Carazzolle, Gonçalo A. G. Pereira, Luiz Filipe P. Pereira, André L. L. Vanzela, Lu Wang, I. King Jordan, Claudia M. A. Carareto

**Affiliations:** 1 Departamento de Biologia, Universidade Estadual Paulista Júlio de Mesquita Filho, University Estadual Paulista, São José do Rio Preto, Brazil; 2 School of Biology, Georgia Institute of Technology, Atlanta, Georgia, United States of America; 3 Departamento de Biologia Geral, Universidade Estadual de Londrina, Londrina, Brazil; 4 Empresa Brasileira de Pesquisa Agropecuária Recursos Genéticos e Biotecnologia (LGM), Brasília, Brazil; 5 Centre de Coopération Internationale en Recherche Agronomique pour le Développement, UMR AGAP, Montpellier, France; 6 Departamento de Genética, Evolução e Bioagentes, Universidade Estadual de Campinas, Campinas, Brazil; 7 Empresa Brasileira de Pesquisa Agropecuária Café, Brasília, Distrito Federal, Brazil; 8 PanAmerican Bioinformatics Institute, Santa Marta, Magdalena, Colombia; National Institutes of Health, United States of America

## Abstract

Plant genomes are massively invaded by transposable elements (TEs), many of which are located near host genes and can thus impact gene expression. In flowering plants, TE expression can be activated (de-repressed) under certain stressful conditions, both biotic and abiotic, as well as by genome stress caused by hybridization. In this study, we examined the effects of these stress agents on TE expression in two diploid species of coffee, *Coffea canephora* and *C. eugenioides*, and their allotetraploid hybrid *C. arabica*. We also explored the relationship of TE repression mechanisms to host gene regulation via the effects of exonized TE sequences. Similar to what has been seen for other plants, overall TE expression levels are low in *Coffea* plant cultivars, consistent with the existence of effective TE repression mechanisms. TE expression patterns are highly dynamic across the species and conditions assayed here are unrelated to their classification at the level of TE class or family. In contrast to previous results, cell culture conditions *per se* do not lead to the de-repression of TE expression in *C. arabica*. Results obtained here indicate that differing plant drought stress levels relate strongly to TE repression mechanisms. TEs tend to be expressed at significantly higher levels in non-irrigated samples for the drought tolerant cultivars but in drought sensitive cultivars the opposite pattern was shown with irrigated samples showing significantly higher TE expression. Thus, TE genome repression mechanisms may be finely tuned to the ideal growth and/or regulatory conditions of the specific plant cultivars in which they are active. Analysis of TE expression levels in cell culture conditions underscored the importance of nonsense-mediated mRNA decay (NMD) pathways in the repression of *Coffea* TEs. These same NMD mechanisms can also regulate plant host gene expression via the repression of genes that bear exonized TE sequences.

## Introduction

Transposable elements (TEs) are genetic entities with an intrinsic mobilization capacity. As a result of this characteristic, they are responsible for donating regulatory sequences [1] and transcription regulatory signals [2], as well as for creating considerable genomic instability, mediating chromosome rearrangements [3], altering both gene expression and function [1], and creating novel genes and exons [4]. Such mobilization can also result in host genome contraction and expansion [5], [6]. According to a unified classification system proposed for eukaryotic transposable elements [7], TEs can be grouped into two classes according to their transposition mode: Class I elements (retrotransposons), which use the enzyme Reverse Transcriptase (RTase) to transpose via an RNA intermediate to a new genome insertion site, and Class II elements, which are transposed directly via DNA molecule using a transposase (Tpase) enzyme. Class I elements are divided into five orders (LTR, DIRS, PLE, LINEs, SINEs), each of which is subdivided into superfamilies (LTR: *Copia*, *Gypsy*, *Bel-Pao*, *Retrovirus*, *ERV*; DIRS: *DIRS*, *Ngaro*, *VIPER*; PLE: *Penelope*; LINEs: *R2*, *RTE*, *Jockey*, *L1*, *I*; SINEs: *tRNA*, *7SL*, *5S*). Class II (DNA transposons) elements are split into two subclasses: subclass I contains superfamilies either with terminal inverted repeats (*Tc1-Mariner*, *hAT*, *Mutator*, *Merlin*, *Transib*, *P*, *PiggyBac*, *PIF-Harbinger* and *Cacta*) or without terminal inverted repeats (*Crypton*), whereas subclass II comprises the Helitron and Maverick superfamilies.

Plant genomes are massively invaded by repetitive DNA, primarily LTR retrotransposons [8]. Many of these retrotransposons are located near host genes and thus could impact the expression of these neighboring genes whose expression is mediated by a variety of mechanisms, such as RNAi [9], DNA methylation [10], and readout transcription [11]. While the transcriptional activity of TEs seems to be tightly controlled by host genomes [12], *e.g.* by transcriptional gene silencing mechanisms such as those that prevent the access of the host transcriptional machinery [13], reports also show that TEs can be activated under certain stress conditions, such as pathogen infection, physical injuries, abiotic stress [14] or cell culture [15], [16], [17], [18].

Of the approximately one hundred species in the *Coffea* genus, only *C. arabica* and *C. canephora* are used in commercial production, representing ∼70% and 30% of global coffee production, respectively [19]. *C. arabica* is a unique polyploidy species of the genus (4n = 4X = 44 chromosomes) and was derived from a recent (1 million years ago) natural hybridization between *C. canephora* and *C. eugenioides* [20]. *C. canephora* is a diploid (2n = 2x = 22 chromosomes) and is an auto-incompatible species that grows in humid and lowland habitats. It is usually more resistant to pests and diseases as well as to abiotic stresses like water deprivation and is also characterized by a higher productivity and bean caffeine content than *C. arabica*. However, the quality of the beverage is regarded as inferior to that of *C. arabica* [21].

The identification of transposable elements in *Coffea* was initiated only recently [22], [23], [24], [25], [26]. To our knowledge, detailed analyses of the abundance, activity and regulation of transcriptionally active TEs in *Coffea* genomes, as well as analyses of the relationship of these to their chromosomal distribution, have yet to be performed. We previously searched the Brazilian Coffee Genome Project database (LGE database, http://www.lge.ibi.unicamp.br/cafe) aiming to identify TE fragments within coding regions in expressed sequences (ESTs) of three *Coffea* species (*C. arabica*, *C. canephora* and *C. racemosa*). The ESTs and unigenes harboring TEs were analyzed regarding the size of the TE fragment, the functional categories to which they were assigned to and to their contribution to the proteome [22]. In the present study, we rescreened the LGE database using a more sensitive method, which allowed for a substantial increase in the number of unigenes harboring TEs, and used the gene sequences to identify paralogous unigenes that do not contain TEs. Expression levels of isoforms with and without TEs in *C. arabica* and *C. canephora* transcriptome were analyzed in cell culture and plants grown under different irrigation conditions. This approach was taken in order to understand the regulatory effects that exonized TEs may exert on *Coffea* host genes. The expression levels of TE transcripts themselves were also assayed across the same conditions in order to better understand how they are regulated and how they respond to various stresses including different drought and irrigation conditions as well as cell culture and polyploidization. The chromosomal distribution of *Coffea* TEs was interrogated genome-wide for the first time here using FISH.

## Results

### Frequency and Classification of Expressed TEs in the Transcriptomes of *Coffea spp*

A set of 181,405 ESTs derived from 39 cDNA libraries (31 from *C. arabica* and 8 from *C. canephora*) were used to identify, classify and quantify the number of expressed TEs. Sequence similarity searches allowed the identification of 320 EST sequences homologous to TEs in the two *Coffea* species (Table 1; Tables S1 & S2 in File S1). For *C. arabica*, the proportion of sequences that were homologous to DNA transposons (51%) and to LTR+NLTR retrotransposons (49%) were similar (*P*>0.05). *Ty3/Gypsy* was the most frequently identified superfamily among the LTR Retrotransposons (22%). However, in *C. canephora,* the proportion of transposons (13.2%) and retrotransposons (86.8%) was considerably different, as were the frequencies of *Ty3/Gypsy* (80.5%) and *Ty1/Copia* (3.6%) (both *P*<0.05).

**Table 1:** Numbers and proportions of ESTs homologous to expressed TEs^1^ in two *Coffea* species.

TEs	Superfamily	*C. arabica ESTs*:N (%)	*C. canephora ESTs*:N (%)
LTR^1^	*Ty1/Copia*	12 (12)	8 (3.6)
	*Ty3/Gypsy*	22 (22)	177 (80.5)
	Not Classified	10 (10)	1 (0.4)
NLTR^2^		5 (5)	5 (2.3)
Total		49 (49.0)	191 (86.8)
Transposons^3^		51 (51.0)	29 (13.2)

1 LTR: LTR Retrotransposons;

2 NLTR Retrotransposons: NLTR;

3 DNA Transposons: Transposons.

**Figure 1:**
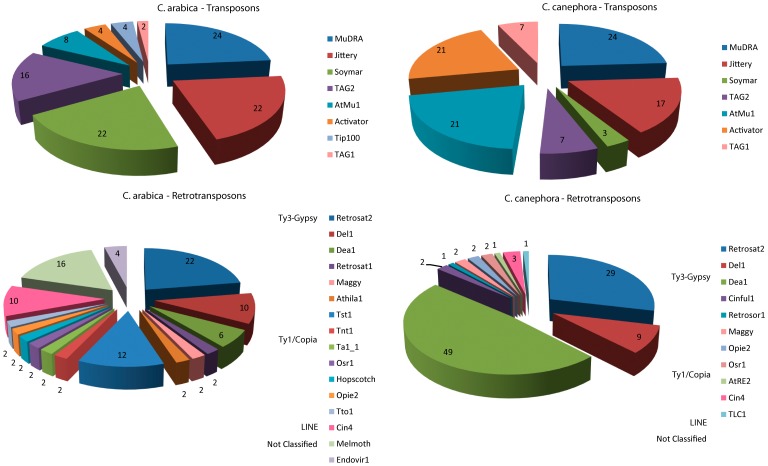
Comparative proportions of distinct TE families in *ESTs* from *C. arabica* and *C. canephora* (data available in Tables S1 and S2 in File S1).

The TEs were classified into families based on the best alignment against a completely characterized reference TE library (Table S3 in File S2). The 100 ESTs from *C. arabica* were classified into 24 families, 8 belonging to DNA transposons and 16 to Retrotransposons (LTR:13, NLTR: 1 and not classified: 2) and the 220 ESTs from *C. canephora* were classified into 18 families (DNA transposons: 7, LTR Retrotransposons: 9, NLTR: 1 and not classified LTR: 1). The main difference between diversity of TE families between the two species is due to higher number of *Ty1-Copia* families in *C. arabica*, which harbors seven families of this superfamily of LTR retrotransposon, but excepting Tst1, all occurring in just one EST (Figure 1, Tables S1 & S2 in File S1). Both species differ also regarding the TEs expression. For example, the most expressed families in *C. arabica* among the retrotransposons, considering their expression among the total TEs and among the TE classes, respectively, were *Retrosat2* (11 ESTs: 11% of the TEs and 22% of Class I), *Melmoth* (8 ESTs: 8% of the TEs and 16% of Class I) and *Tst1* (6 ESTs: 6% of the TEs and 12% of Class I). Regarding the DNA transposons, the most expressed families in *C. arabica* were *MuDR* (12 ESTs: 12% of the TEs and 24% of Class II), Jittery (11 ESTs: 11% of the TEs and 22% of Class II) and *Soymar* (11 ESTs: 11% of the TEs and 22% of Class II). Likewise, in *C. canephora* the most expressed retrotransposons were *dea1* (94 ESTs: 43% and 49%), *Retrosat2* (56 ESTs: 25% and 29%) and *Del1* (18 ESTs: 8% and 9%) and, the most expressed DNA transposons were *MuDR* (7 ESTs: 3% and 24%), *AtMu* and *Activator* (6 ESTs each: 2.7% and 21%), and *Jittery* (5 ESTs: 2% and 17%).

### Characterization of cDNA Clones from *C. arabica* Similar to TEs

After the 100 clones identified in *C. arabica* were classified into families, the redundant clones (identical ESTs and identified in the same library) were excluded and 64 clones remained, from which a sample of 27 were fully sequenced (Table S4 in File S3) and the remaining partially sequenced. Again, this set of sequences was compared against two TE banks: the reference elements cited above and the collection of consensus TEs stored in Repbase [27]. The results showed that these cDNAs were highly similar to elements from Repbase described in species closely related to the genus *Coffea*, such as *Vitis vinifera* and *Solanum tuberosum*. The occurrence of frame shifts and stop codons was distinct for some comparisons due to the differential choice of frame in the sequence translation. Putative complete transposase or polyprotein searches were performed by evaluating CDSs that spanned at least 60% of the reference or consensus TE and had no frame shifts and stop codons.

### Expression Levels of TE Transcripts

**Figure 2:**
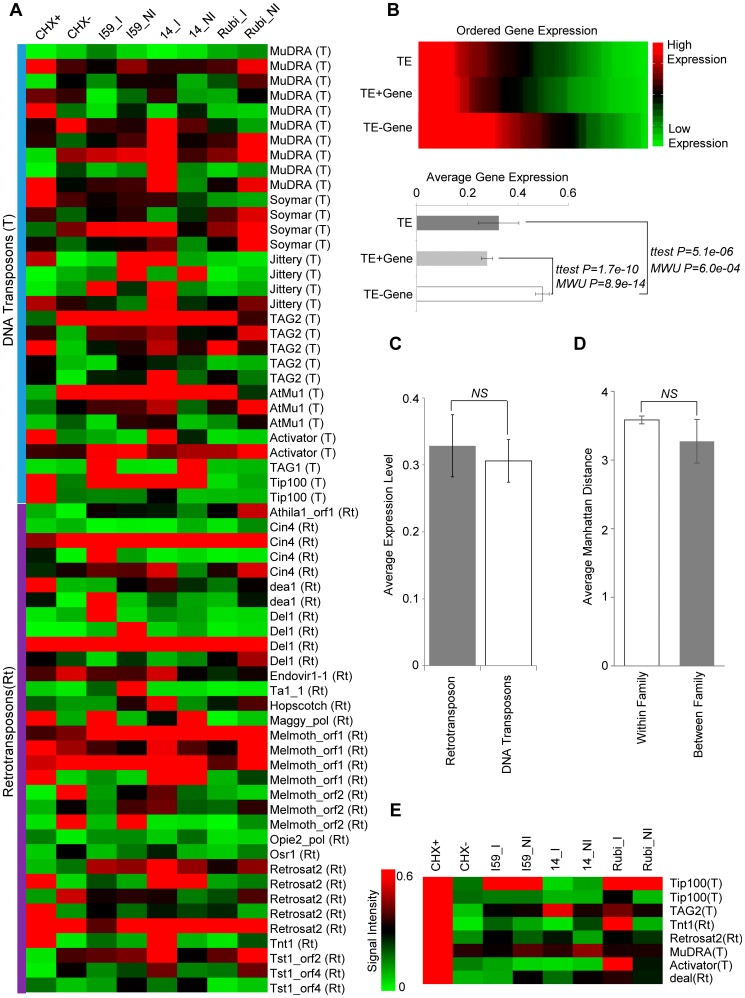
Expression levels of TE transcripts. (A) Heatmap showing the relative expression levels of TE transcripts for 31 DNA transposons and 33 retrotransposons. **CHX+:** *C. arabica* callus treated with cycloheximide, **CHX–:** *C. arabica* callus untreated, **I59_I**: *C. arabica* irrigated leaves from drought tolerant cultivar Iapar59, **I59_NI**: *C. arabica* non-irrigated leaves from Iapar59, **14_I**: *C. canephora* irrigated leaves from drought tolerant cultivar, **14_NI**: *C. canephora* non-irrigated leaves from drought tolerant cultivar, **Rubi_I**: *C. arabica* irrigated leaves from drought sensitive cultivar Rubi, **Rubi_NI**: *C. arabica* non-irrigated leaves from Rubi. (B) Overall expression level differences between TEs, genes with TE insertions (TE+Genes, *n = 77*) and genes without TE insertions (TE–Genes, *n = 63*) across the conditions measured here. Average expression levels ± standard errors were compared using the Students’ t-test and the Mann-Whitney U test (MWU) as indicated. (C) Average expression levels for retrotransposons versus DNA transposons. (D) Average Manhattan distances between expression profiles within versus between TE families. (E) Individual TE sequences that have significantly up-regulated upon cycloheximide treatment (CHX+).

Macroarray expression profiling was performed for 64 TE transcripts (31 DNA transposons and 33 retrotransposons) in six samples from the allotetraploid *C. arabica* (callus treated with cycloheximide versus untreated callus (CHX+, CHX–), irrigated versus non-irrigated leaves (_I, _NI) from drought tolerant versus drought sensitive cultivars (I59, Rubi)), and in two samples from the diploid *C. canephora* (14_). Many TEs exhibit relatively low expression levels (Figure 2A and Figure S1 in File S4), and overall TEs are expressed at significantly lower levels than genes measured for the same cultivars and conditions (Figure 2B).

TE expression patterns across the species, cultivars and conditions assayed here are highly dynamic and apparently unrelated to their classification at the level of TE class (DNA transposon versus retrotransposons) or at level of specific TE families (Figure 2C). When hierarchical clustering is used to relate the expression patterns of individual TE transcripts, there is no apparent coherence within TE classes or families; individual members of TE classes and families are dispersed throughout the resulting tree (Figure S2 in File S4). The coherence of expression patterns within and between TE families was also measured using average Manhattan distances between TE expression profiles. There is no statistical difference in the average distances between TE expression profiles within or between families (Figure 2D). This same lack of overall coherence in TE expression patterns can be seen at the level of individual TE families, where there is generally no difference in the distances between expression profiles within or between families (Figure S3 in File S4). Exceptions to this overall pattern can be seen for the Jittery and Tip100 families of DNA transposons, which have relatively coherent within family expression patterns (Figure 2A and Figure S3 in File S4). Interestingly, TE expression for CHX– (callus untreated) is not higher than that of the other tissues/conditions in *C. arabica* (Figure S4 in File S4), suggesting that cell culture conditions do not de-repress TE expression in this species.

**Figure 3:**
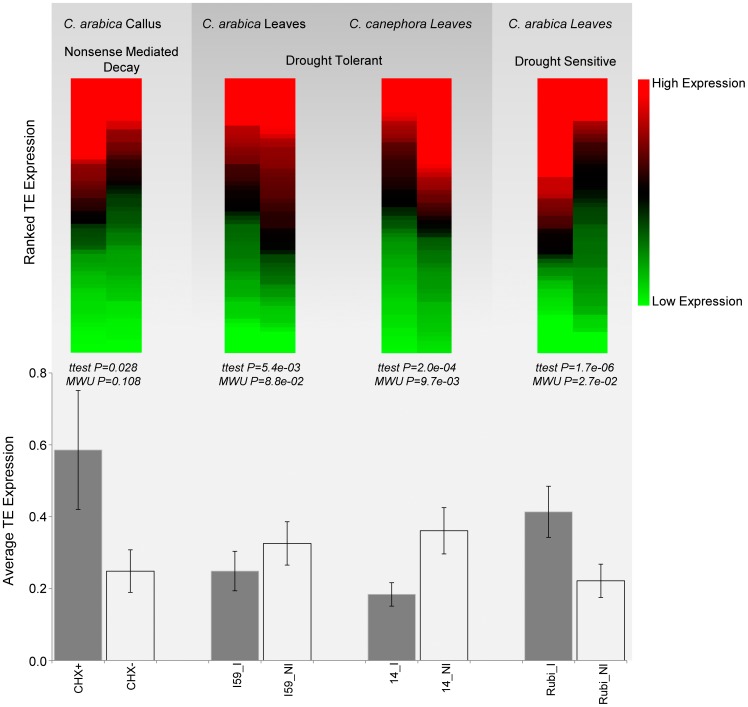
TE expression level differences for paired cultivar samples. Overall TE expression levels are compared for cycloheximide treated (CHX+) versus untreated (CHX–) *C. arabica* callus and irrigated (I) versus non-irrigated (NI) leaves for drought tolerant *C. arabica* and *C. canephora* as well as drought sensitive *C. arabica*. Average expression levels ± standard errors were compared using the Students’ t -test and the Mann-Whitney U test (MWU) as indicated.

The dynamic expression patterns seen for individual TE transcripts, along with the overall lack of TE expression pattern coherence within classes and families of elements, suggest that the expression of individual TE insertions is independently regulated based in part on the surrounding genomic environment. This may include both sequence-based and epigenetic factors for the surrounding genomic environment. However, it should be noted that changes in TE expression in response to cycloheximide treatment appear to be more consistent across individual TE insertions. On average, TEs are expressed at higher levels in callus treated with cycloheximide (CHX+ versus CHX– in Figure 3); although, the difference is only marginally significant owing to the high level of variation in TE expression under cycloheximide treatment. Nevertheless, there are a number of individual TE transcripts that show highly significant differences between CHX+ and CHX– conditions (Figure 2E and Figure S5 in File S4). Cycloheximide (CHX) is a compound that blocks translation and thereby inactivates the nonsense-mediated mRNA decay pathway (NMD). The NMD pathway provides for the detection and degradation of aberrant mRNAs that contain premature termination codons resulting from point mutations, rearrangements or errors during transcription or RNA processing [28]. More to the point, NMD may also represent a genome defense mechanism against the proliferation of TEs since mRNA sequences derived from TEs are frequent targets of NMD [29]. Thus, the up-regulation of TE expression upon CHX treatment would seem to reflect the mitigation of the plant genome’s NMD based defense against TE expression. The consistency of this pattern seen across TE transcripts assayed here underscores the likely importance of NMD in genome defense against plant TE expression.

We performed similar comparative analyses in order to evaluate whether there are overall differences in TE expression between paired samples of irrigated (I) and non-irrigated (NI) cultivars that are either drought sensitive or drought tolerant. TE transcripts tend to be expressed at significantly higher levels in non-irrigated cultivars for the drought tolerant samples; this effect is marginally significant for *C. arabica* leaves and more highly significant for *C. canephora* leaves (Figure 3). Drought sensitive cultivars from *C. arabica* show the opposite pattern with irrigated samples showing significantly higher TE expression (Figure 3). Considered together, these results suggest the interesting possibility that TE expression levels may be tuned to the drought response proclivities of their host genomes.

### TE–Derived CDSs

The unigenes containing TE cassettes (i.e. exonized TEs) previously identified in the *C. arabica* and *C. canephora* ESTs using RepeatMasker [22] are presented in Tables S5 & S6 in File S5, respectively. Each unigene cluster contains sequences that represent a unique gene resulting from assembling of various ESTs. Rescreening the ESTs of *C.arabica* against the library of 2,503 consensus TEs from RepBase yielded 421 TE–containing ESTs. Due to the high number of matches, only those with E-values<e^−10^ were analyzed, resulting in 303 matches. A comparison of these TE–containing ESTs against the database of unigenes (EST clusters) in the LGE database revealed 27 new TE–derived protein CDSs in *C. arabica* (Table S5 in File S5). All of the CDSs matched with proteins with a clearly defined function, namely protein factors related to transcriptional and spliceosomal machinery, chaperones and alcohol dehydrogenase.

### Effect of Exonized TEs on the Expression and Regulation of TE–containing Transcripts by NMD

Screening the unigenes containing TE cassettes from *C. arabica* (86) and *C. canephora* (59) against their respective sets of unigenes allowed the identification of 111 and 47 paralogous unigenes, respectively, based on their high sequence similarity (Tables S5 & S6 in File S5), as illustrated in Figure S6 (File S4). Two sets of alignments showed evidence of putative alternative splicing in *C. arabica*. The first had three transcripts (uni_CA_046, uni_CA_125 and uni_CA_127) that were similar to a rust resistance Rp1-D-like protein (GB: AAK18308) and the second had two transcripts (uni_CA_055 and uni_CA_138) that were similar to a universal stress protein (USP) family protein (GB: NP_850562). Additionally, this analysis is not exhaustive because 1) the EST libraries were built based only on the representation of the 5′ end of mRNAs, preventing the identification of TEs in other portions of the transcript; 2) several libraries were not targeted for full sequencing (saturation), particularly for *C. canephora,* which meant that fewer tissues were represented and fewer ESTs were obtained; and 3) the limited *Coffea* genomic resources do not enable a complete analysis of gene structure and TE insertion sites or the identification of transcripts related to each expressed locus.

**Figure 4:**
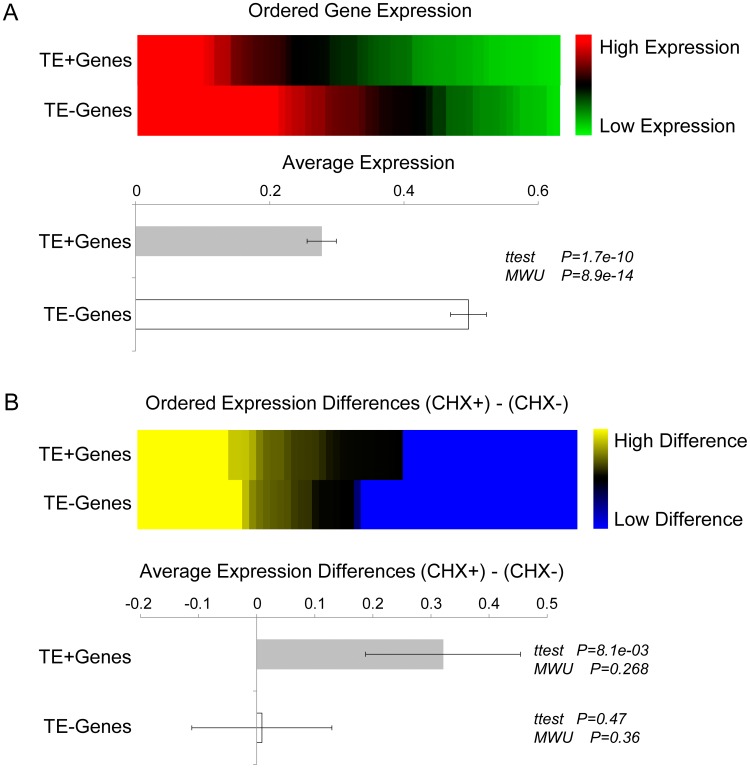
Effect exonized TEs on gene expression. (A) Comparison of overall expression levels of genes with TE cassettes (TE+Genes, *n* = 77) versus genes with no TE cassettes (TE–Genes, *n* = 63). Average expression levels ± standard errors were compared using the Students’ t-test and the Mann-Whitney U test (MWU) as indicated. (B) Differences in overall expression levels between CHX+ and CHX– conditions for TE+Genes versus TE–Genes. Average expression levels ± standard errors were compared between CHX+ and CHX– conditions for TE+Genes and TE–Genes individually using the Students’ t-test and the Mann-Whitney U test (MWU) as indicated.

The expression levels of 77 gene transcripts containing TE cassettes (TE+Genes) and 63 paralogous gene transcripts that lacked TEs (TE–Genes) were analyzed using macroarrays across the same eight cultivars as previously described for *C. arabica* and *C. canephora* (Figure S1 in File S4). TE+Genes have significantly lower levels of overall expression than TE–Genes (Figure 4A & Figure S7A in File S4), and in fact TE+Gene expression levels are not distinguishable from those of TE transcripts themselves (Figure 2B). This result indicates that the presence of TEs in genic transcripts leads to their down-regulation and suggests the possibility that mechanisms similar to those used to repress TE transcript, such as NMD, may be involved in this process. To test this possibility, we compared the effects of CHX treatment on TE+Genes versus TE–Genes. TE+Genes show significantly greater overall levels of expression in CHX+ conditions compared to CHX–, whereas TE–Genes show no such difference in expression across the different CHX treatments (Figure 4B & Figure S7B in File S4). CHX+ conditions, which are seen facilitate the expression of TE+Genes, inactivate the NMD pathway for aberrant transcript repression. Thus, these results are consistent with the activation of the NMD pathway by the presence of TE cassettes within expressed gene transcripts.

### Chromosomal Distribution of TEs in *C. arabica* and its Parental Species

**Figure 5:**
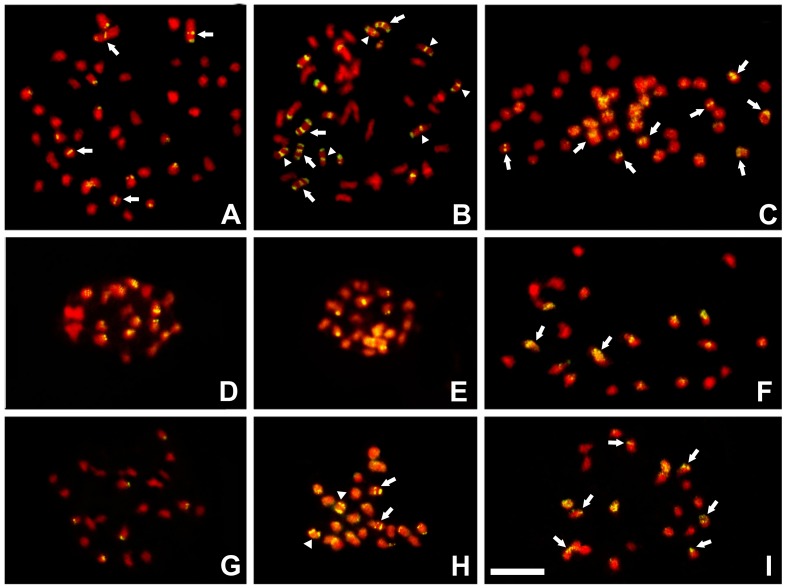
Chromosomal locations of TEs. FISH using sequences of transposons *MuDRA* (GI311206994), *Tip100* (GI 315896428) and of retrotransposon *Del1* (GI 315862857) in the chromosomes of *C. arabica* var. *typica*, *C*. *eugenioides* and *C*. *canephora*. The *MuDRA* probe hybridized in 14 locations in *C. arabica* var. *typica* (**A**), with terminal, interstitial and proximal signals. Arrows indicate interstitial/proximal sites. This same probe hybridized preferentially clustered signals in *C*. *eugenioides*, with scattered signals in two pairs (**D**) and only clustered terminal signals in *C*. *canephora* (**G**). The *Tip100* probe showed 36 hybridization sites in *C. arabica* var. *typica* (**B**), with chromosomes containing three sites (arrows) and two hybridization sites in terminal and proximal/interstitial regions (arrowheads). The same probe showed only eight chromosomes with terminal sites in *C*. *eugenioides* (**E**) and 14 chromosomes with signals in *C*. *canephora* (**H**). Note that four chromosomes exhibit two signals, being terminal and interstitial (arrows) and double terminal (arrowheads). The *Del1* probe hybridized in 20 chromosomes in *C. arabica* var. *typica* (**C**). In only eight of them clustered signals were observed (arrows). From 12 chromosomes with signals observed in *C. eugenioides* (**F**), only two presented scattered ones (arrows). For *C*. *canephora* (**I**), this probe showed two pairs with scattered signals and evident terminal signals in six chromosomes (arrows). Bar represents 10 µm.

Three TEs (two transposons: *MuDR* and *Tip100* and one LTR-retroelement: *Del*1) with average low expression levels were selected, and their chromosomal distribution was evaluated using FISH in *C. arabica* var. *typica*, *C*. *eugenioides* and *C*. *canephora* (Figure 5). The transposon signals were preferentially clustered in chromosomal terminal positions in the two ancestors (*C*. *eugenioides* and *C*. *canephora*) of *C. arabica*. Interstitial and/or proximal signals were observed in larger numbers in *C. arabica* var. *typica*, especially using the *Tip100* probe, indicating an increase in transposition activity in this species compared to the parental species.

The probe containing the LTR-retrotransposon *Del*1 showed a different hybridization profile, exhibiting signals that were more scattered. The number of positions hybridized with the *Del*1 probe in *C. arabica* was nearly the same as that of the sum of signals of *C. eugenioides* and *C*. *canephora*. Furthermore, *C. canephora* chromosomes showed preferentially clustered signals, and most *C. eugenioides* chromosomes showed dispersed signals. These results are in agreement with previous FISH results [30] using another probe containing a fragment homologous to a gag-like element from a *Del*1 LTR-retrotransposon isolated from the germplasm of *C. arabica var. typica*. Similarities in *Ty*1-*copia*-like retroelement among the different *Coffea* genomes were also reported [25].

## Discussion

This study provides a preliminary understanding of the TE regulatory dynamics in the allotetraploid and complex genome of *C. arabica*. The findings presented here show that this species is an interesting study organism because most DNA transposons and retrotransposons seem to be submitted to fine transcriptional control. Differences and similarities with other plant genomes were observed. For instance, it has been reported that cell culture conditions increase the expression levels of some TEs in plants, as for example *Tnt1* from tobacco [31]; *Tos10, Tos17 and Tos19* from rice [32]; several DNA transposons and retrotransposons from sugarcane [17]; and LTR retrotransposons from rice [18]. In contrast to these previous observations, our results show that cell culture conditions *per se* do not elevate TE expression levels in *C. arabica*. On average, TEs in untreated cell culture conditions (CHX–) show no significant difference in expression levels compared to TEs analyzed from *C. arabica* plant tissue (Figure S4 in File S4). These results suggest that TE regulatory and/or suppression mechanisms remain largely intact in *C. arabica* cell culture conditions. This conclusion is supported by the observation that treatment of *C. arabica* cell culture with CHX, a repressor of NMD, leads to a significant increase in TE expression (Figure 3). In other words, the NMD regulatory systems that suppress TE expression remain active in *C. arabica* cell culture.

Results reported here also underscore the importance of NMD-related TE repression systems for the regulation of host genes. Indeed, NMD not only represses the expression of the TEs themselves (Figure 3) but also appears to repress the expression of host genes that contain exonized TE sequences (Figure 4). This finding represents a novel molecular mechanism by which TE sequences can influence the regulation of *Coffea* host gens.

Polyploid genomes are subjected to extensive changes, such as insertions/deletions, inversions and translocations, as well as alterations in gene expression patterns [33], [34]. Although the mechanisms of these changes are poorly understood, increasing TE transpositional activity is a possibility since quiescent TEs in a diploid genome can become activated in the new polyploid genetic environment. Additionally, the genetic redundancy in a polyploid genome can mitigate the deleterious effects of transposition [35], thereby allowing TEs to proliferate in allotetraploids and insert within gene-rich chromosomal regions. Another possibility is the relaxation of host silencing mechanisms (*e.g.*, methylation) in allotetraploids, which should also allow for increased transposition rates [36]. These factors may explain the increase in transposon copy number and their more prevalent interstitial chromosomal location we observed in the allotetraploid *C. arabica* compared to its parents, *C. canephora* and *C. eugenioides*.

*Coffea arabica* and *C. canephora* showed a low TE–like mRNA abundance; only 0.17% of ESTs were expressed TEs (320 out of 181,405 *EST*s). This low abundance has also been observed in other plant genomes. For an example, 60% of the *Z. mays* genome is composed of retroelements, but only 0.014% of these retroelements (56 out of 407,000 ESTs) were identified as expressed [37]. However, a recent systematic search in the maize transcriptome showed that 1.5% of its ESTs (25,282 ESTs out of more than 2 million) were similar to 56 well characterized TE families [18]. In *Saccharum officinarum*, out of 260,781 ESTs, 276 (0.1%) were considered to be expressed TEs [38]. Finally, in *Eucalyptus grandis*, out of 123,889 ESTs, 124 (0.1%) were identified as transcriptionally active TEs [39]. Our data reinforce the fact that TEs are poorly represented in the *Coffea* transcriptome, although plant genomes are enriched by those repetitive sequences. This paradox reflects the strong host repression of TE transcriptional and transpositional activity in plants, as is illustrated by the paucity of TEs in *Coffea* transcriptomes and the heterochromatic distribution of most *Coffea* TE sequences.

Nevertheless, transcriptional activation of several plant retrotransposons under stress has been shown and it seems that these mobile sequences have adapted to their host genomes through the evolution of highly regulated promoters that mimic those of the stress-induced plant genes (see [14], for a review). Moreover, it has been also shown, as for example with Tnt1 in tobacco, that subfamilies of the same retrotransposon show different stress-associated patterns of expression [40]. Here, in a broader analysis of TE expression, we demonstrate that differing plant drought stress levels relate strongly to the changes in TE expression levels observed upon changes in irrigation conditions. Drought stress conditions were evaluated here in terms of predawn leaf water potentials *Ψ*_pd_. In coffee (as in many other plants), *Ψ*_pd_ values close to 0 (>−0.4 MPa) are observed for unstressed plants, while more negative values (<−0.4 MPa) characterize drought stress. In other words, more highly negative *Ψ*_pd_ values reflect higher levels of drought stress. Thus, the observed *Ψ*_pd_ value of −0.80 MPa for *C.arabica* cv. Iapar59 is considered to show moderate drought stress, whereas the −3.02 MPa *Ψ*_pd_ value of *C. canephora* clone 14 is considered to show severe drought stress [41], [42]. Both of these drought tolerant *Coffea* cultivars show higher expression in the non-irrigated conditions (Figure 3). Interestingly, the opposite pattern was observed for drought sensitive cultivars from *C. arabica*, which showing significantly higher levels of TE expression in irrigated conditions (Figure 3). Given the presumed effects of genomic environment on the expression of individual TE transcripts noted above, these divergent phenomena may relate to the overall state of the particular plant genome with respect to its ideal drought-related growth and regulatory conditions. In drought sensitive plants, TE expression levels go down in drought conditions consistent with an overall depression of genomic regulatory activity. On other hand, TE expression is up-regulated upon drought conditions in drought tolerant plants presumably consistent with the ideal growth/regulatory conditions of these cultivars.

## Materials and Methods

The ESTs used in our study are derived from libraries of the Brazilian Coffee Genome Project, hereafter called PGCB (http://www.lge.ibi.unicamp.br/cafe), which contain partial sequences of cDNA of a wide range of tissues (*e.g.*, seeds, embryogenic calli, roots, leaves, flowers), developmental stages and plant material submitted to biotic (e.g., stems infected with *Xylella spp* and nematodes) and abiotic (*e.g.*, water deficit) stress conditions [43], [22]. They comprises 131,150 ESTs from thirty-one cDNA libraries of *C. arabica* and 50,255 ESTs from eight cDNA libraries of *C. canephora* (12,332 obtained from 2 Brazilian Coffee Genome Project libraries plus 37,923 of six libraries from Lin et al. 2005 [44], deposited at the SOL Genomics Network http://www.sgn.cornell.edu/content/coffee.pl). These ESTs were analyzed with two main objectives: (1) to characterize the classes, types and numbers of expressed TEs, and to investigate their expression; and 2) to investigate the impact of fragments of TEs (TE–cassettes) inserted in coding regions of both species comparing the expression of sequences harboring TE–cassettes and homologous sequences not harboring TE–cassettes (likely paralogous sequences).

### Construction of a Permanent cDNA Library of Clones of Interest

To characterize the expression profiles of active TEs and to evaluate putative differences in expression of transcripts containing TE–cassettes compared to their isoforms without TEs, the 242 cDNA clones of interest (64 of expressed TEs, 86 of unigenes with TE–cassettes and their 77 of isoforms without TEs) were trimmed from the PGCB libraries. Multiplication of 3 µL of bacteria culture containing each cDNA insert, cryopreserved in glycerol (50% v/v) and kept at −80°C was allowed in deepwell plates with 1.2 ml LB liquid culture medium for. After growth, 75 µL was removed to construct a permanent library with only the cultures of interest, followed by the purification and cloning of DNA plasmids.

### Identification of Expressed TEs

Expressed TEs were identified in 181,405 ESTs (libraries of *C. arabica* plus of *C. canephora*). The transcripts were considered likely to represent a transcriptionally active element when the TE sequence occupied more than 70% of an EST or unigene. EST clusters that were similar to expressed TEs were not considered because they may represent the mRNA assemblies of distinct insertions. Searches for the transcriptionally active TEs were performed using keywords such as “transposon”, “transposase”, “polyprotein”, “retrotransposon”, “retroposon”, “MITEs”, “LINEs”, “SINEs” and family names (e.g., “hAT”, “MuDR”, “En/Spm”) of the *ESTs*. A BLASTx [45] comparison of the *ESTs* with TE annotations against the protein sequences in the NCBI NR (non-redundant) database was then performed. Many BLAST hits were obtained. To eliminate spurious and unreliable results, a stringent cut-off (E = 1e^−30^) was applied. The resulting transcripts were classified into families according to the best alignment by BLASTx or tBLASTx [45] against a completely characterized library of 96 reference TEs (Table S3 in File S2), as well as against 840 consensus TEs of phylogenetic species close to *Coffea* (e.g., *Vitis vinifera*, *Populus trichocarpa*, *Solanum lycopersicum*, and *Solanum tuberosum*) obtained from the dicotyledonous plant library Repbase (www.girinst.com). When ESTs or unigene sequences were annotated by the alignment with more than one reference TE from different plant TE databases, the matches between *Coffea* transcripts and reference TEs with the higher RM score was chosen. The frequencies of retrotransposons and transposons, as well as expressed TEs of the *Ty-Copia* and *Ty3-Gypsy* superfamilies were compared using a χ^2^ test.

From the set of 100 clones homologous to TEs of *C. arabica* (Table S1 in File S1), 64 were cloned into pSPORT1 vector, and sequenced using the *BigDye® Terminator v3.1 Cycle Sequencing kit* (Applied Biosystems, Foster City, CA, USA) using the universal primers M13F (5′-GTAAAACGACGGCCAG-3′) and M13R (5′-CAGGAAACAGCTATGAC-3′) as well as through internal primers specific to each clone and run on a *3730xl DNA Sequencer* (Applied Biosystems). The sequences were clustered using CodonCode Aligner v.3.5.6 (www.codoncode.com), and bases had a Phred quality ≥20. The identification notation of these active TEs was “Ca_” (for *C. arabica*), “TE–” (for transposable elements) plus “three numerical digits”, for example Ca_TE_031. Full sequences were obtained for 27 of these clones and partial sequences (sizes over 50% of the total length) for the remaining 37.

### Identification of Novel Cases of TEs Incorporated into Mature mRNAs from *C. arabica* and of Paralogous Sequences without TEs

It has been shown that the choice of sequence similarity search methods to detect TE–derived sequences strongly influences the estimate of TE-cassettes that can be identified in protein coding regions [46]. Hidden Markov model based searches followed by BLAST methods (tBLASTx → tBLASTn → BLASTx → BLASTp → BLASTn) and RepeatMasker are more sensitive in identifying exonized TEs. We used that protocol to identify novel TE–derived sequences in protein coding sequences of *Coffea* in addition to those previously identified by RepeatMasker alone [22]. A total of 131,150 ESTs from *C. arabica* were compared by tBLASTx [45] against 2,503 plant consensus TEs from Repbase [27]. To avoid spurious results, only the best E≤e^−10^ matches were accepted, without imposing additional scores or length thresholds. ESTs containing TEs were then compared to EST clusters of each species for the identification of their respective unigenes. They summed 145 cDNA clones containing TE–cassettes (59 from *C. arabica* obtained in our previous study [22] plus 27 novel ones obtained in this study, and 59 from *C. canephora*), which were compared by BLASTn [45] against all cDNA clones of each species. This procedure allowed the identification of highly similar and thus likely paralogous unigenes without TEs. Examples of alignments between unigenes containing TEs and the highly related unigenes using sequence similarity searches by BLASTn are given in Figure S6 (File S4).

### Expression Analyses

The expression analyses were carried out for the 64 individual transcriptionally active TEs characterized in this study (Table 2) and for transcripts of CDSs harboring TE–cassettes (77) identified in this and in a previous study [22] and corresponding CDSs without TE insertions (63), identified in this study (Table 3).

**Table 2:** List of CDSs similar to expressed TE families identified in the transcriptome from *C. arabica* used as target in the macroarray analyses using as probe RNA samples from *C. arabica*.

Query id	Library	Subject id	GenBank Accession
Ca_TE–001	RM1	MuDRA	GW476772.1
Ca_TE–003	IC1	MuDRA	GW461848.1
Ca_TE–004	IA2	MuDRA	GW460883.1
Ca_TE–005	EA1	MuDRA	GW439358.1
Ca_TE–006	CS1	MuDRA	GT724977.1
Ca_TE–007	SH2	MuDRA	GW447279.1
Ca_TE–008	LV5	MuDRA	GT697838.1
Ca_TE–009	FB1	MuDRA	GT709698.1
Ca_TE–011	CA1	MuDRA	GT688551.1
Ca_TE–012	FR1	MuDRA	GT714837.1
Ca_TE–015	RT8	Jittery	GW451071.1
Ca_TE–017	LV8	Jittery	GW478609.1
Ca_TE–018	EA1	Jittery	GW445953.1
Ca_TE–019	FB1	Jittery	GW480270.1
Ca_TE–025	SI3	Soymar	GT720097.1
Ca_TE–030	LV4	Soymar	GT694144.1
Ca_TE–031	LV4	Soymar	GT694146.1
Ca_TE–033	CS1	Soymar	GT724651.1
Ca_TE–036	RX1	TAG2	GW444348.1
Ca_TE–037	LV4	TAG2	GW488918.1
Ca_TE–038	RT8	TAG2	GW452630.1
Ca_TE–039	LV5	TAG2	GW470411.1
Ca_TE–042	FR2	TAG2	GW468343.1
Ca_TE–043	SH2	AtMu1	GW446952.1
Ca_TE–045	IC1	AtMu1	GT731348.1
Ca_TE–046	CB1	AtMu1	GW460044.1
Ca_TE–047	PA1	Activator_orf1	GT685618.1
Ca_TE–048	LV4	Activator_orf2	GW465099.1
Ca_TE–049	FB2	Tip100	GW463960.1
Ca_TE–050	SH2	Tip100	GW447257.1
Ca_TE–051	SI3	TAG1	GW432669.1
Ca_TE–053	LV8	Retrosat2	GW470427.1
Ca_TE–057	BP1	Retrosat2	GW436442.1
Ca_TE–059	FB1	Retrosat2	GW481089.1
Ca_TE–061	SH2	Retrosat2	GW447231.1
Ca_TE–062	RT5	Retrosat2	GT686160.1
Ca_TE–063	FR2	Cin4	GW467887.1
Ca_TE–064	RT8	Cin4	GW429899.1
Ca_TE–065	PC1	Cin4	GT671271.1
Ca_TE–066	FR1	Cin4	GW487483.1
Ca_TE–068	FB2	Melmoth_orf1	GW485897.1
Ca_TE–069	FR1	Melmoth_orf1	GW473493.1
Ca_TE–071	BP1	Melmoth_orf1	GW436111.1
Ca_TE–072	CL2	Melmoth_orf1	GT678668.1
Ca_TE–073	LV5	Del1	GW469064.1
Ca_TE–075	RM1	Del1	GW476916.1
Ca_TE–076	IC1	Del1	GW434887.1
Ca_TE–077	FR1	Del1	GW472574.1
Ca_TE–079	LV8	dea1	GW470679.1
Ca_TE–080	RT5	dea1	GT686341.1
Ca_TE–081	FR1	Tst1_orf4	GW473442.1
Ca_TE–082	LV5	Tst1_orf4	GW469004.1
Ca_TE–085	CA1	Tst1_orf2	GT689576.1
Ca_TE–086	SH2	Tst1_orf2	GW447114.1
Ca_TE–088	CB1	Melmoth_orf2	GW458400.1
Ca_TE–089	CL2	Melmoth_orf2	GT680947.1
Ca_TE–090	PA1	Endovir1-1	GT684931.1
Ca_TE–093	FR1	Tnt1	GW473549.1
Ca_TE–094	CL2	Ta1_1_rt	GT681881.1
Ca_TE–095	CB1	Osr1	GW428435.1
Ca_TE–096	LV4	Athila1_orf1	GW465397.1
Ca_TE–097	EA1	Hopscotch	GW439671.1
Ca_TE–098	CA1	Opie2_pol	GT688707.1
Ca_TE–100	FB1	Maggy_pol	GW474059.1

**Query id:** arbitrary identification; Ca_TE–001 - Ca_TE–059: DNA Transposons; Ca_TE–060 - Ca_TE–100: Retrotransposons; **Library**: tissue, developmental stage or stress condition in which the clone was obtained (BP1 - Suspension cells treated with acibenzolar-S-methyl, CA1 - Non-embryogenic callus, CB1 - Suspension cells treated with acibenzolar-S-methyl and brassinosteroids, CL2 - Hypocotyls treated with acibenzolar-S-methyl, FB1 - Flower buds in stages 1 and 2–long, FR1 - Flower buds no 6, pinhead fruits no 1 and fruits (stages 1 and 2)–long, FR2 - Flower buds no 6, pinhead fruits no 1 and fruits (stages 1 and 2)–short, LV4 - Young leaves from orthotropic branch – long, LV5 - Young leaves from orthotropic branch–short, PA1 - Primary embryogenic callus, RT5 - Roots with acibenzolar-S-methyl, RT8 - Suspension cells stressed with aluminum, SH2 - Water deficit stresses plants (pool of tissues).

**Table 3:** List of unigenes containing or not TE–cassette insertions identified in the transcriptome from *C. arabica* used as target in the macroarray analyses using as probe RNA samples from *C. arabica*.

Unigenes containing TEs insertions				Unigenes related those containing TE by BLASTn comparisons			
Query id	Library	First protein hit in BLASTx searches	GenBank Accession	Query id	Library	First protein hit in BLASTx searches	GenBank Accession
uni_CA_003	CB1	no hits	GW429042.1	uni_CA_061	CB1	no hits	GW429023.1
uni_CA_004	CB1	Calreticulin 1 precursor	GW460351.1	uni_CA_064	RX1	known protein	GW444133.2
uni_CA_005	CS1	DRL1 (deformed roots and leaves 1)	GW431410.1	uni_CA_066	CL2	no hits	GT679138.1
uni_CA_008	EA1	probable kinesin heavy chain	GW439522.1	uni_CA_074	FB2	known protein	GW485670.1
uni_CA_009	EA1	no hits	GW439324.1	uni_CA_076	CA1	no hits	GT687716.1
uni_CA_010	FB1	no hits	GW481560.1	uni_CA_079	CB1	no hits	GW459902.1
uni_CA_011	FB2	no hits	GW463979.1	uni_CA_080	RT8	ubiquitin	GT727921.1
uni_CA_012	FB2	known protein	GW463474.1	uni_CA_081	LV5	polyubiquitin	GT696191.1
uni_CA_013	FB2	no hits	GT702329.1	uni_CA_082	LV5	pentameric ubiquitin	GW468856.1
uni_CA_014	FB2	known protein	GW464459.1	uni_CA_084	LV5	ubiquitin	GW469836.1
uni_CA_015	FB4	known protein	GW481999.1	uni_CA_085	SH2	ubiquitin	GW446992.1
uni_CA_016	FB4	known protein	GW462957.1	uni_CA_086	RT8	hexameric polyubiquitin	GT727889.1
uni_CA_017	LV4	Ser/Thr protein kinase	GW465620.1	uni_CA_087	LV5	polyubiquitin	GW469698.1
uni_CA_018	LV5	no hits	GW469849.1	uni_CA_088	CB1	hexameric polyubiquitin	GT734282.1
uni_CA_019	LV9	no hits	GT712029.1	uni_CA_089	FR1	ubiquitin	GW473010.1
uni_CA_021	RT8	aldo/keto reductase family	GW429915.1	uni_CA_090	IC1	polyubiquitin	GT731354.1
uni_CA_022	SH2	no hits	GW447711.1	uni_CA_091	FR1	polyubiquitin	GT714268.1
uni_CA_023	SH2	cell wall-plasma membrane linker protein	GW441504.1	uni_CA_092	RM1	polyubiquitin	GT709959.1
uni_CA_024	SH2	known protein	GW446464.1	uni_CA_093	LV5	polyubiquitin	GW470359.1
uni_CA_025	SI3	no hits	GW434416.1	uni_CA_095		polyubiquitin	GT729693.1
uni_CA_026	SI3	no hits	GW456069.1	uni_CA_096	BP1	polyubiquitin	GW454486.1
uni_CA_027	CL2	no hits	GT679363.1	uni_CA_097	LV8	polyubiquitin	GW477703.1
uni_CA_029	RT8	no hits	GW450760.1	uni_CA_098	FB1	polyubiquitin	GW475362.1
uni_CA_031	CL2	fertility restorer	GT681814.1	uni_CA_099	RX1	heavy-metal-associated domain-containing protein	GW443677.1
uni_CA_032	FR2	known protein	GW490007.1	uni_CA_101	CA1	known protein	GT690863.1
uni_CA_033	FB2	transfactor-like	GW485664.1	uni_CA_102	CS1	known protein	GW432153.1
uni_CA_034	LV8	heavy-metal-associated domain-containing protein	GW478558.1	uni_CA_103	CL2	no hits found/rab GDP dissociation inhibitor	GT679591.1
uni_CA_036	FB4	rab GDP dissociation inhibitor	GT713090.1	uni_CA_104	LV8	no hits found/PSTVd RNA-binding protein Virp1a	GW472348.1
uni_CA_037	LV8	putative Ruv DNA-helicase	GW470488.1	uni_CA_105	CL2	no hits found/PSTVd RNA-binding protein Virp1a	GT679878.1
uni_CA_038	LV5	PSTVd RNA-binding protein Virp1a	GT695351.1	uni_CA_108	FB2	glyceraldehyde-3-phosphate dehydrogenase, cytosolic	GW485790.1
uni_CA_039	FB4	calreticulin precursor	GT712569.1	uni_CA_109	FB2	glyceraldehyde-3-phosphate dehydrogenase, cytosolic	GW485584.1
uni_CA_040	PC1	multidomain cyclophilin type peptidyl-prolyl cis-trans isomerase - CYP63	GT671168.1	uni_CA_111	FB4	calreticulin 2	GW463324.1
uni_CA_041	LV9	SRG1 (senescence-related gene 1), oxidoreductase	GT711910.1	uni_CA_113	CS1	no hits	GW449610.1
uni_CA_042	FB4	ribosomal protein L7	GT713207.1	uni_CA_114	RM1	multidomain cyclophilin type peptidyl-prolyl cis-trans isomerase	GW483167.1
uni_CA_043	LV8	ubiquitinating enzyme	GT699245.1	uni_CA_116	CA1	SRG1 (senescence-related gene 1), oxidoreductase	GT688114.1
uni_CA_044	LV8	no hits	GW471330.1	uni_CA_117	BP1	SRG1 (senescence-related gene 1), oxidoreductase	GW436064.1
uni_CA_046	CA1	rust resistance Rp1-D-like protein	GT691135.1	uni_CA_119	PC1	SRG1 homolog	GT669760.1
uni_CA_047	FR2	pre-mRNA splicing factor cwc15/Cwc15 cell cycle control	GW467203.1	uni_CA_120	PC1	SRG1 like protein	GT670925.1
uni_CA_048	LV5	CONSTANS-like protein	GW492003.1	uni_CA_121	IA2	SRG1 like protein	GT715475.1
uni_CA_049	AR1	no hits	GT695192.1	uni_CA_122	CB1	ribosomal protein L7	GT733345.1
uni_CA_051	LV8	PSTVd RNA-binding protein Virp1a	GT698901.1	uni_CA_123	LV8	ribosomal protein L7	GW477964.1
uni_CA_052	RM1	no hits	GW476842.1	uni_CA_124	RT8	ribosomal protein L7	GT727945.1
uni_CA_053	LV5	transmembrane MLO family protein	GW469299.1	uni_CA_125	LV5	NBS-LRR type resistance protein	GW491220.1
uni_CA_054	SI3	known protein	GW456436.1	uni_CA_126	IC1	NBS-LRR type resistance protein	GW462143.1
uni_CA_055	SI3	universal stress protein (USP) family protein	GW456437.1	uni_CA_127	CA1	disease resistant protein rga4	GT689692.1
uni_CA_056	SI3	known protein	GW456352.1	uni_CA_128	FR1	Vrga1	GW473126.1
uni_CA_057	SH2	known protein	GW447523.1	uni_CA_131	CL2	no hits found/sucrose synthase	GT681626.1
uni_CA_058	SH2	no hits	GT717519.1	uni_CA_132	CL2	no hits found/sucrose synthase	GT681063.1
uni_CA_059	RX1	putative SET protein, phospatase 2A inhibitor/nucleosome assembly protein	GT730810.1	uni_CA_133	RM1	PSTVd RNA-binding protein Virp1a	GW476706.1
uni_CA_071	IA2	Putative Cer1	GT715250.1	uni_CA_135	FR1	no hits	GW473180.1
uni_CA_072	FB2	Putative Cer1	GW463949.1	uni_CA_136	CA1	transmembrane MLO family protein	GT687779.1
uni_CA_073	FB1	no hits	GT707977.1	uni_CA_137	CA1	transmembrane MLO protein family	GT687778.1
uni_CA_075	AR1	no hits	GT695018.1	uni_CA_139	FR1	nucleosome/chromatin assembly factor A	GW488045.1
uni_CA_077	SH2	no hits	GW440861.1	uni_CA_145	RT8	SC35-like putative splicing factor	GW429957.1
uni_CA_078	CB1	no hits	GW459862.1	uni_CA_156	CA1	EIL3	GT688815.1
uni_CA_115	LV8	SRG1 (senescence-related gene 1), oxidoreductase	GW479323.1	uni_CA_168	RM1	SC35	GW483810.1
uni_CA_140	SI3	galactokinase GHMP kinase-like	GW433589.1	uni_CA_170	LP1	heat shock cognate protein 70	GT672528.1
uni_CA_141	FB2	GHMP kinase-like protein	GW464703.1	uni_CA_174	CL2	heat shock protein	GT676556.1
uni_CA_142	FB2	SC35-like putative splicing factor	GW464703.1	uni_CA_175	FR1	molecular chaperone Nthsp70	GW486995.1
uni_CA_143	EA1	SC35-like putative splicing factor	GW439909.1	uni_CA_181	LV8	HSP70 luminal binding protein precursor	GT699444.1
uni_CA_144	RM1	SC35-like putative splicing factor	GW476757.1	uni_CA_182	LV5	Luminal binding protein 5 precursor	GW491798.1
uni_CA_145	RT8	SC35-like putative splicing factor	GW429957.1	uni_CA_183	LV8	heat shock protein 70	GW472345.1
uni_CA_146	CS1	SC35-like splicing factor	GT724318.1	uni_CA_185	LV5	EIN3-like protein	GT696422.1
uni_CA_147	FB1	SC35-like putative splicing factor	GW475083.1	uni_CA_186	RT8	EIL2	GT727361.1
uni_CA_149	LV4	protein F21D18.16	GW488672.1	uni_CA_188	LV9	EIN3-like protein	GT711462.1
uni_CA_150	BP1	heat shock cognate 70 kd protein	GW454864.1	uni_CA_189	IA2	expressed protein	GW461009.1
uni_CA_151	LV4	dnaK-type molecular chaperone hsp70	GT694219.1	uni_CA_193	SH2	no hits found/putative Ruv DNA-helicase	GT717446.1
uni_CA_153	FR1	heat shock cognate protein 70	GW472995.1	uni_CA_194	FB1	putative Ruv DNA-helicase	GT709711.1
uni_CA_154	BP1	heat shock cognate protein 70	GT722106.1	uni_CA_195	SH2	putative Ruv DNA-helicase	GW441596.1
uni_CA_155	CA1	EIL3	GT688815.1	uni_CA_196	NS1	putative Ruv DNA-helicase	GT686944.1
uni_CA_156	SI3	EIL3	GT718525.1	uni_CA_197	LV4	putative Ruv DNA-helicase	GW488879.1
uni_CA_157	RT8	EIL2	GT727360.1	uni_CA_198	FB1	RNA Binding Protein 47	GT709653.1
uni_CA_158	CS1	expressed protein	GW432395.1	uni_CA_199	LV9	Cwf15-Cwc15 cell cycle control protein	GW486969.1
uni_CA_159	CL2	histone H3.2	GT674334.1	uni_CA_200	FB2	putative CEO protein	GT701573.1
uni_CA_161	LV5	histone	GW469621.1	uni_CA_201	FB4	putative CEO protein (29126336)	GW482151.1
uni_CA_162	BP1	histone H3	GT722310.1	uni_CA_202	RT8	ceo protein	GT727214.1
uni_CA_163	FB2	histone H3.2	GT701769.1	uni_CA_206	SI3	putative SCO1 protein	GW455770.1
uni_CA_164	SI3	putative Ruv DNA-helicase	GW457317.1				
uni_CA_165	CB1	putative Ruv DNA-helicase	GW459457.1				
uni_CA_166	SH2	histone H3	GW446727.1				
uni_CA_167	SI3	SC35-like splicing factor	GW433455.1				
uni_CA_190	LV8	histone H3	GT701173.1				
uni_CA_192	EA1	histone H3	GW439934.1				
uni_CA_203	BP1	cell wall-plasma membrane linker protein	GT721229.1				
uni_CA_204	LV8	putative nascent polypeptide associated complex alpha chain	GW478999.1				
uni_CA_205	FB1	putative nascent polypeptide associated complex alpha chain	GT708359.1				

* **Query id:** arbitrary identification by RepeatMasker and tBLASTx; **Library**: tissue, developmental stage or stress condition in which the clone was obtained (BP1 - Suspension cells treated with acibenzolar-S-methyl, CA1 - Non-embryogenic callus, CB1 - Suspension cells treated with acibenzolar-S-methyl and brassinosteroids, CL2 - Hypocotyls treated with acibenzolar-S-methyl, FB1 - Flower buds in stages 1 and 2–long, FR1 - Flower buds no 6, pinhead fruits no 1 and fruits (stages 1 and 2)–long, FR2 - Flower buds no 6, pinhead fruits no 1 and fruits (stages 1 and 2)–short, LV4 - Young leaves from orthotropic branch – long, LV5 - Young leaves from orthotropic branch–short, PA1 - Primary embryogenic callus, RT5 - Roots with acibenzolar-S-methyl, RT8 - Suspension cells stressed with aluminum, SH2 - Water deficit stresses plants (pool of tissues).

#### Plant material

For probe synthesis, total mRNA was extracted from the following samples of *C. arabica:* a) Drought stress: leaves of cultivars tolerant (Iapar59) and sensitive (Rubi) to drought grown in field conditions (Cerrado Agricultural Research Center, Planaltina-DF, Brazil) with (predawn leaf water potentials *Ψ*_pd_−0.38±0.10 and −0.22±0.07 MPa for Iapar59 and Rubi cultivars, respectively) and without (*Ψ*_pd_ = −0.80±0.12 and −1.88±0.36 MPa for Iapar59 and Rubi cultivars, respectively) irrigation [47], b) Cell culture: embryogenic callus from *C. arabica* cv. Catuaí Vermelho maintained in a multiplication medium for ∼4 months; c) Inhibition treatment: the same embryogenic callus was treated for 4 h with the protein biosynthesis inhibitor cycloheximide (CHX: 10 and 30 mg/mL in alcohol) added to cell culture for a final concentration of 100 and 300 µg/mL. *C. canephora* var. conilon drought stress: clone 14, tolerant to drought was selected by the INCAPER [41] and grown in a greenhouse with (unstressed condition, *Ψ*_pd_ leaves = −0.02±0.03 MPa) or without (stress, *Ψ*_pd_ leaves = −3.02±0.12 MPa) water [42]. For fluorescent in situ hybridization (FISH), slides were prepared with root tips of *C. arabica* var. typica, *C. canephora* and *C. eugenioides* pretreated with a saturated solution of paradichlorobenzene for 24 h at 14°C, without acid hydrolysis.

#### RNA isolation, DNAse treatment and reverse transcription

RNA of all samples was extracted from cells using Concert™ reagent (Invitrogen, Carlsbad, CA, USA) according to the manufacturer’s protocol. Total RNA samples (10 µg) were incubated for 30 min at 37°C with 3 U of *RQ1 RNAse-Free DNAse* (Promega, Madison, WI, USA) in a final volume of ∼10 µl. Each total RNA sample was mixed with 1.5 µl Oligo(dT)_12–18_ (Invitrogen), heated at 75°C for 10 min, and then cooled for 5 min on ice. The reaction mixture for the reverse transcription contained 5 µl of 5×first-strand buffer, 2.5 µl of 0.1 mol/l DTT, 40 U of RNAseOUT, 50 µCi (α-^33^P)-dCTP and 2.5 µl of a 10 mM mixture of unlabeled dNTPs (dATP, dTTP and dGTP) and was heated at 42°C for 5 min. The reverse transcription was performed at 42°C for 20 min with 300 U of *SuperScript III First-Strand Synthesis System for RT-PCR* (Invitrogen). Then, 1.25 µl of unlabeled dCTP (10 mM) was added and maintained for 1 h, terminated by heating at 94°C for 5 min and cooled for 5 min on ice. The total volume (30.25 µl) was used in the hybridization experiments.

#### Amplification of target DNA

Each target cDNA (100 ng) was amplified by PCR in a volume of 25 µl with 1.25 U of *Platinum® Taq DNA Polymerase* (Invitrogen) in 10× polymerase buffer, 2 mM MgCl_2_, 200 µM each dNTP and 10 µM of each universal primer, M13F and M13R. The solutions were heated to 94°C for 2 min, followed by 35 cycles of denaturation (94°C for 30 sec), annealing (50°C for 30 sec), extension (72°C for 4 min), and final extension at 72°C for 7 min. The target DNAs were used to make the membrane arrays.

#### Macroarray experiments and analysis

The PCR products of the target DNAs were denatured in DMSO (50% v/v) for 30 min at 37°C, arrayed in a 384-well microtiter plate and then spotted twice in the same position onto *Performa II nylon filters* (Genetix Limited, Hampshire, UK) using the high-throughput robot system *Q-BOT* (Genetix Limited). To increase the signal homogeneity among spots and filters, the set of 64 cDNAs was spotted in duplicate (2×2 array) onto two identical arrays using the same nylon filters (222×222 mm). Additionally, 16 spots containing cDNA of the reference gene ubiquitin were applied to delimit the two arrays. After sample deposition, the filters were dampened with a denaturant solution (NaCl 0.13 M and 0.5 M NaOH) for 10 min and a neutralization solution (NaCl 1.5 M and Tris 1 M) for 5 min, then fixed by UV light exposition (1,200 µj/cm^2^) for 12 sec and stored at −80°C. The filters were pre-hybridized for 2 h at 65°C in *Modified Church and Gilbert Buffer* (0.5 M Na Phosphate Buffer pH 7.2, 7% SDS, 10 mM EDTA) and hybridized overnight with cDNA sample probes. Membranes were washed for 15 min three times with 0.1% SDS/1×SSC and three times with 0.1% SDS/0.1×SSC at 65°C. After washing, the filters were exposed on imaging plates *BAS-MS 2340* (Fujifilm, Tokyo, Japan) for 72 h in a *BAS 2340 cassette* (Fujifilm) and scanned using a fluorescent image analyzer *FLA3000* (Fujifilm). The radioactive intensity of each spot was quantified by *Array Gauge* software (Fujifilm), corrected by the level of the local background, normalized to the average intensities of the reference gene ubiquitin (except for callus treated with CHX, in which the reference gene expression was completely suppressed). For differences in probe labeling, normalization was by use of the average signals of all genes studied. The homogeneity of the spot replicates were evaluated and represented by average values using *limma* of the Bioconductor package [48] from R (http://www.r-project.org).

The resulting normalized expression levels for individual probes, expressed as signal intensity values, were visualized, clustered and statistically analyzed across all conditions assayed here. Signal intensity values of individual transcripts were visualized and hierarchically clustered using the TIGR Multiexperiment viewer (MeV) program (http://www.tm4.org/mev.html). Average expression levels between conditions were compared using both parametric (Student’s ttest) and non-parametric (Mann-Whitney U test) statistical tests. Differences in condition-specific expression profiles for individual transcripts were computed using Manhattan distances between signal intensity vectors across conditions. The resulting distances were averaged within and between TE classes and families to measure TE expression coherence.

### Fluorescent *in Situ* Hybridization

FISH was performed as described elsewhere [49] with modifications. Three expressed TE cDNA clones (GI 311206994, GI 315896428 and GI 315862857 similar to *MuDR*, *Tip100* and *del1,* respectively) were used to synthesize the probes with biotin-14-dATP by nick translation. The reaction mixture (total volume 33 µl) contained 15 µl of 100% formamide, 6 µl of polyethylene glycol, 3 µl of 20×SSC, 1 µl of calf thymus DNA (100 ng), 4 µl of water and 4 µl of each probe (200 ng). The samples were denatured at 70°C for 10 min, and hybridization was performed at 37°C overnight in a humidified chamber. The washes were carried out in 6×SSC and 4×SSC/0.2% Tween 20 at room temperature. The probes were detected with avidin-FITC, followed by post-detection washes in 4×SSC/0.2% Tween 20 at room temperature. Slides were mounted with 25 µl of antifade, composed of glycerol (90%), 1,4-diaza-bicyclo(2,2,2)-octane(2.3%), 20 mM Tris-HCl pH 8.0 (2%), water and 1 µl of 2 µg/ml 4,6-diamidino-2-phenylindole (DAPI). The images were acquired using a *Leica DM4500 B Microscope* (Leica Microsystems, Wetzlar, Germany) equipped with a *DFC 300FX Digital Color Camera* (Leica Microsystems), and the image was overlapped with red color for DAPI using the *Leica IM50 4.0* image management software (Leica Microsystems).

## Supporting Information

File S1 **Tables S1 & Table S2.** List of CDSs similar to expressed TE families identified in the transcriptome from *C. arabica* (Table S1) and *C. canephora* (Table S2).
(PDF)

File S2 **Table S3.** Completely characterized transposable elements library used for the classification into families of the expressed TEs identified in the *Coffea* transcriptome.
(PDF)

File S3 **Table S4:** List of fully cloned 27 cDNA from *Coffea arabica* similar to plant TEs.
(PDF)

File S4 **Figures S1–S7.** Supplementary Figures for the TE and gene expression analyses.
(PDF)

File S5 **Tables S5 & Table S6.** List of unigenes containing or not TE–cassette insertions in *C. arabica* (Table S5) and *C. canephora* (Table S6).
(PDF)
