# Supplementary material for: Transcriptional Activity, Chromosomal Distribution and Expression Effects of Transposable Elements in Coffea Genomes
Source: PLoS One. 2013 Nov 11;8(11):e78931. doi: 10.1371/journal.pone.0078931 (PMC3823963; doi:10.1371/journal.pone.0078931)
Supplement: File S1 — Tables S1 & Table S2. List of CDSs similar to expressed TE families identified in the transcriptome from C. arabica (Table S1) and C. canephora (Table S2). (PDF) [file pone.0078931.s001.pdf]

FILE S1

Transcriptional activity, chromosomal distribution and expression effects of transposable elements in Coffea genomes

Fabrício R. Lopes[1,+], Daudi Jjingo[2,+], Carlos R. M. da Silva[3], Alan C. Andrade[4], Pierre Marraccini[4,5], João B. Teixeira[4], Marcelo F. Carazzolle[6], Gonçalo A. G. Pereira[6], Luiz Filipe P. Pereira[7], André L.L. Vanzela[3], Lu Wang[2], I. King Jordan[2,8] and Claudia M. A. Carareto*1

[1] Departamento de Biologia, UNESP , Univ. Estadual Paulista, São José do Rio Preto, SP, Brazil

[2]School of Biology, Georgia Institute of Technology, Atlanta, USA

[3] Departamento de Biologia Geral, Universidade Estadual de Londrina, Londrina, PR, Brazil

[4] EMBRAPA Recursos Genéticos e Biotecnologia (LGM), Brasília, DF, Brazil

[5]CIRAD, UMR AGAP, Montpellier, France

[6] Departamento de Genética, Evolução e Bioagentes, Universidade Estadual de Campinas, Campinas, SP, Brazil

[7]EMBRAPA Café, Brasília, Distrito Federal, Brazil

[8]PanAmerican Bioinformatics Institute, Santa Marta, Magdalena, Colombia

[+]These authors contributed equally to the work

*Corresponding author (phone: 55 17 3221 2382; fax 55 17 3221 2390; e-mail carareto@ibilce.unesp.br)

Table S1. List of CDSs similar to expressed TE families identified in the transcriptome from *C. arabica*.

Query id	Library	Subject id	% identity	alignment length	mis-matches	gap openings	query start	query end	subject start	subject end	e-value	bit score	GenBank Accession
**DNA Transposons**													
Ca_TE-001	RM1	MuDRA	27.00	100	72	1	394	690	118	217	2,00E-06	42.4	GW476772.1
Ca_TE-002	SI3	MuDRA	29.90	194	132	2	190	759	133	326	2,00E-23	99.4	GW433300.1
Ca_TE-003	IC1	MuDRA	39.71	68	40	1	433	633	136	203	3,00E-10	55.1	GW461848.1
Ca_TE-004	IA2	MuDRA	28.91	128	90	1	398	778	136	263	2,00E-16	75.5	GW460883.1
Ca_TE-005	EA1	MuDRA	30.36	247	169	2	137	868	136	382	5,00E-36	140	GW439358.1
Ca_TE-006	CS1	MuDRA	29.38	194	132	4	89	655	146	339	7,00E-22	94.0	GT724977.1
Ca_TE-007	SH2	MuDRA	33.33	264	173	3	99	881	165	424	6,00E-43	164	GW447279.1
Ca_TE-008	LV5	MuDRA	29.92	254	173	4	32	778	178	431	4,00E-31	124	GT697838.1
Ca_TE-009	FB1	MuDRA	29.50	200	140	3	114	710	310	497	2,00E-22	95.9	GT709698.1
Ca_TE-010	FB1	MuDRA	23.43	239	181	5	69	779	337	563	2,00E-17	79.3	GW480953.1
Ca_TE-011	CA1	MuDRA	26.54	260	186	4	59	823	343	590	2,00E-23	99.4	GT688551.1
Ca_TE-012	FR1	MuDRA	26.11	180	132	3	70	606	343	510	8,00E-20	70.9	GT714837.1
Ca_TE-013	FR1	Jittery	28.83	111	67	2	8	304	40	148	5,00E-10	54.7	GW487058.1
Ca_TE-014	SI3	Jittery	28.40	257	150	6	96	764	52	304	4,00E-28	107	GW455169.1
Ca_TE-015	RT8	Jittery	29.53	298	181	5	77	883	92	388	7,00E-38	147	GW451071.1
Ca_TE-016	LV8	Jittery	38.25	251	155	3	59	811	142	389	2,00E-45	171	GW478796.1
Ca_TE-017	LV8	Jittery	37.97	266	161	3	76	861	142	403	3,00E-50	188	GW478609.1
Ca_TE-018	EA1	Jittery	36.70	188	119	0	300	863	194	381	2,00E-38	149	GW445953.1
Ca_TE-019	FB1	Jittery	35.09	285	181	2	53	895	267	550	6,00E-50	187	GW480270.1
Ca_TE-020	FB1	Jittery	32.71	321	199	4	52	963	267	572	3,00E-49	185	GW481620.1
Ca_TE-021	LV8	Jittery	35.92	142	90	2	77	499	298	431	3,00E-28	85.9	GW479380.1
Ca_TE-022	SI3	Jittery	34.88	86	55	1	208	462	538	623	1,00E-08	50.1	GW433113.1
Ca_TE-023	SI3	Jittery	34.88	86	55	1	206	460	538	623	1,00E-08	50.1	GW456204.1
Ca_TE-024	SI3	Soymar	45.83	168	91	2	279	782	22	184	1,00E-40	156	GT720098.1
Ca_TE-025	SI3	Soymar	47.49	179	94	2	357	893	22	195	6,00E-44	167	GT720097.1
Ca_TE-026	SI3	Soymar	55.94	261	115	3	105	887	46	300	1,00E-80	288	GT720100.1
Ca_TE-027	SI3	Soymar	56.28	183	80	2	96	644	50	227	1,00E-66	206	GT720099.1
Ca_TE-028	SI3	Soymar	57.00	200	86	1	109	708	79	276	8,00E-74	238	GT720101.1
Ca_TE-029	SH2	Soymar	58.15	270	112	3	90	896	106	372	2,00E-86	308	GT717659.1
Ca_TE-030	LV4	Soymar	57.71	175	74	1	52	576	153	326	1,00E-55	202	GT694144.1

Table S1. Continuation.

Ca_TE-031	LV4	Soymar	58.11	265	111	1	58	852	153	416	3,00E-87	311	GT694146.1
Ca_TE-032	LV4	Soymar	58.70	247	102	1	54	794	153	398	3,00E-81	291	GT694145.1
Ca_TE-033	CS1	Soymar	60.30	199	79	1	63	659	180	377	9,00E-65	236	GT724651.1
Ca_TE-034	SI3	Soymar	59.82	112	45	1	407	742	266	376	3,00E-35	132	GT720102.1
Ca_TE-035	CB1	TAG2	39.02	205	122	5	108	713	65	264	2,00E-32	129	GW428372.1
Ca_TE-036	RX1	TAG2	46.72	244	129	2	824	96	94	335	4,00E-61	224	GW444348.1
Ca_TE-037	LV4	TAG2	32.68	205	136	2	80	688	110	311	4,00E-34	127	GW488918.1
Ca_TE-038	RT8	TAG2	44.02	209	117	0	50	676	194	402	4,00E-49	184	GW452630.1
Ca_TE-039	LV5	TAG2	31.36	287	188	8	94	927	252	516	8,00E-26	107	GW470411.1
Ca_TE-040	LV5	TAG2	40.96	83	49	0	528	280	253	335	1,00E-33	77.0	GW492555.1
Ca_TE-041	FR2	TAG2	25.13	195	144	3	63	641	300	479	9,00E-23	72.0	GW467844.1
Ca_TE-042	FR2	TAG2	29.80	255	177	3	72	830	300	539	3,00E-32	128	GW468343.1
Ca_TE-043	SH2	AtMu1	29.48	173	118	3	92	598	266	436	3,00E-22	95.1	GW446952.1
Ca_TE-044	LV4	AtMu1	24.18	244	171	3	97	786	451	693	3,00E-15	72.0	GW488420.1
Ca_TE-045	IC1	AtMu1	28.46	123	87	1	97	462	491	613	7,00E-09	50.8	GT731348.1
Ca_TE-046	CB1	AtMu1	28.47	144	83	3	166	537	556	699	2,00E-11	59.3	GW460044.1
Ca_TE-047	PA1	Activator_orf1	32.03	153	103	2	277	732	54	204	3,00E-28	81.3	GT685618.1
Ca_TE-048	LV4	Activator_orf2	25.74	202	136	5	278	841	12	188	3,00E-14	55.8	GW465099.1
Ca_TE-049	FB2	Tip100	52.06	194	90	2	309	881	380	569	2,00E-48	182	GW463960.1
Ca_TE-050	SH2	Tip100	59.55	178	72	0	689	156	383	560	6,00E-55	203	GW447257.1
Ca_TE-051	SI3	TAG1	23.53	238	156	8	137	772	22	252	6,00E-15	70.9	GW432669.1
**Retrotransposons**													
Ca_TE-052	LV8	Retrosat2	53.95	215	99	0	23	667	631	845	2,E-71	251	GW470733.1
Ca_TE-053	LV8	Retrosat2	53.11	241	113	0	57	779	633	873	3,E-75	271	GW470427.1
Ca_TE-054	LV8	Retrosat2	50.62	243	120	0	57	785	633	875	4,E-70	253	GW472073.1
Ca_TE-055	LV8	Retrosat2	52.70	241	114	0	56	778	633	873	1,E-76	270	GW477675.1
Ca_TE-056	FB1	Retrosat2	35.55	211	135	2	57	686	813	995	3,E-31	125	GW474619.1
Ca_TE-057	BP1	Retrosat2	28.28	244	150	4	227	883	891	1131	4,E-35	98.6	GW436442.1
Ca_TE-058	BP1	Retrosat2	40.00	105	63	0	263	577	891	995	5,E-30	84.0	GW453951.1
Ca_TE-059	FB1	Retrosat2	36.40	250	149	6	762	43	1246	1487	1,E-36	142	GW481089.1

Table S1. Continuation.

Ca_TE-060	FB1	Retrosat2	29.84	248	164	6	769	56	1248	1487	1,E-22	**96.3**	GW481133.1
Ca_TE-061	SH2	Retrosat2	34.08	179	109	5	135	644	1344	1521	9,E-17	**77.0**	GW447231.1
Ca_TE-062	RT5	Retrosat2	31.37	102	67	2	74	370	1365	1466	7,E-09	**50.8**	GT686160.1
Ca_TE-063	FR2	Cin4	34.74	190	123	3	276	842	456	636	2,E-26	109	GW467887.1
Ca_TE-064	RT8	Cin4	35.98	164	101	3	566	87	528	685	7,E-27	91.3	GW429899.1
Ca_TE-065	PC1	Cin4	35.56	180	113	2	101	631	729	907	1,E-19	86.7	GT671271.1
Ca_TE-066	FR1	Cin4	31.03	87	60	0	69	329	1030	1116	5,E-08	48.1	GW487483.1
Ca_TE-067	FR1	Cin4	31.03	87	60	0	69	329	1030	1116	5,E-08	48.1	GW472540.1
Ca_TE-068	FB2	Melmoth_orf1	36.32	234	149	3	56	757	13	237	6,E-41	156	GW485897.1
Ca_TE-069	FR1	Melmoth_orf1	25.97	181	130	2	333	863	21	197	2,E-19	85.9	GW473493.1
Ca_TE-070	FR1	Melmoth_orf1	25.71	175	126	2	339	851	21	191	2,E-18	82.4	GW473171.1
Ca_TE-071	BP1	Melmoth_orf1	25.33	225	157	5	61	702	55	277	2,E-16	76.3	GW436111.1
Ca_TE-072	CL2	Melmoth_orf1	44.16	77	43	1	358	588	543	618	6,E-18	80.9	GT678668.1
Ca_TE-073	LV5	Del1	32.75	171	114	2	930	421	66	235	1,E-20	90.1	GW469064.1
Ca_TE-074	PA1	Del1	32.35	102	69	0	422	117	134	235	5,E-12	61.2	GT684690.1
Ca_TE-075	RM1	Del1	20.42	191	132	6	120	632	255	432	4,E-06	41.6	GW476916.1
Ca_TE-076	IC1	Del1	51.02	49	24	1	514	660	1189	1236	7,E-08	47.4	GW434887.1
Ca_TE-077	FR1	Del1	42.86	175	100	1	777	253	1268	1441	4,E-42	150	GW472574.1
Ca_TE-078	LV8	dea1	67.03	182	60	0	40	585	225	406	1,E-68	249	GW478926.1
Ca_TE-079	LV8	dea1	60.70	257	97	2	54	812	225	481	5,E-83	296	GW470679.1
Ca_TE-080	RT5	dea1	48.75	80	41	0	312	73	613	692	2,E-20	89.0	GT686341.1
Ca_TE-081	FR1	Tst1_orf4	72.60	146	40	0	233	670	1	146	2,E-64	227	GW473442.1
Ca_TE-082	LV5	Tst1_orf4	62.02	208	79	2	84	707	100	305	6,E-75	264	GW469004.1
Ca_TE-083	CA1	Tst1_orf3	38.34	193	114	3	920	357	202	392	8,E-32	127	GT690878.1
Ca_TE-084	SH2	Tst1_orf3	48.57	35	18	0	156	260	358	392	2,E-06	42.7	GW441569.1
Ca_TE-085	CA1	Tst1_orf2	33.91	230	133	7	56	688	52	260	2,E-24	102	GT689576.1
Ca_TE-086	SH2	Tst1_orf2	40.91	66	37	1	662	853	263	328	1,E-09	53.1	GW447114.1

Table S1. Continuation.

Ca_TE-087	SH2	Melmoth_orf2	53.37	193	90	0	97	675	40	232	4,E-59	217	GW447731.1
Ca_TE-088	CB1	Melmoth_orf2	53.50	157	73	0	38	508	88	244	8,E-45	170	GW458400.1
Ca_TE-089	CL2	Melmoth_orf2	44.83	87	48	0	76	336	160	246	1,E-16	76.6	GT680947.1
Ca_TE-090	PA1	Endovir1-1	38.60	57	35	0	572	742	950	1006	2,E-15	48.5	GT684931.1
Ca_TE-091	EA1	Endovir1-1	48.72	156	80	0	579	112	1301	1456	5,E-44	149	GW439527.1
Ca_TE-092	PA1	Retrosat1	41.46	41	24	0	500	378	788	828	1,E-09	43.9	GT685739.1
Ca_TE-093	FR1	Tnt1	48.00	50	26	0	127	276	1277	1326	7,E-11	57.4	GW473549.1
Ca_TE-094	CL2	Ta1_1_rt	61.86	118	45	0	39	392	449	566	9,E-39	150	GT681881.1
Ca_TE-095	CB1	Osr1	24.12	199	139	3	309	869	46	239	3,E-19	85.1	GW428435.1
Ca_TE-096	LV4	Athila1_orf1	41.62	197	115	0	647	57	52	248	4,E-44	167	GW465397.1
Ca_TE-097	EA1	Hopscotch	54.03	124	57	0	354	725	922	1045	3,E-39	140	GW439671.1
Ca_TE-098	CA1	Opie2_pol	22.87	188	142	6	94	648	61	232	2,E-08	40.4	GT688707.1
Ca_TE-099	CA1	Tto1	22.93	205	157	7	68	679	334	517	6,E-10	54.3	GT690975.1
Ca_TE-100	FB1	Maggy_pol	24.27	206	123	5	62	580	564	769	1,E-16	55.5	GW474059.1

Query id: arbitrary identification; Library: tissue, developmental stage or stress condition in which the clone was obtained (BP1 - Suspension cells treated with acibenzolar-S-methyl, CA1 - Non-embryogenic callus, CB1 - Suspension cells treated with acibenzolar-S-methyl and brassinosteroids, CL2 - Hypocotyls treated with acibenzolar-S-methyl, FB1 - Flower buds in stages 1 and 2—long, FR1 - Flower buds no 6, pinhead fruits no 1 and fruits (stages 1 and 2)—long, FR2 - Flower buds no 6, pinhead fruits no 1 and fruits (stages 1 and 2)—short, LV4 - Young leaves from orthotropic branch — long, LV5 - Young leaves from orthotropic branch—short, PA1 - Primary embryogenic callus, RT5 - Roots with acibenzolar-S-methyl, RT8 - Suspension cells stressed with aluminum, SH2 - Water deficit stresses plants (pool of tissues); **Subject id:** identification of the reference TEs (Table S3 in File S2).

Table S2. List of CDSs similar to expressed TE families identified in the transcriptome from *C. canephora*.

Query id	Library	Subject id	% identity	alignment length	mis-matches	gap openings	q. start	q. end	s. start	s. end	e-value	bit score	GenBank Accession
**DNA Transposons**													
CC_TE-001	SE2	MuDRA	30.04	243	165	5	4	717	138	379	1,E-26	110	DV706100.1
CC_TE-002	SE4	MuDRA	28.33	180	104	7	505	41	171	347	7,E-15	70.9	DV703680.1
CC_TE-003	SE4	MuDRA	27.08	192	105	8	475	5	171	359	2,E-14	69.3	DV694299.1
CC_TE-004	SE4	MuDRA	28.33	180	104	7	469	5	171	347	9,E-15	70.9	DV703680.1
CC_TE-005	SE4	MuDRA	28.33	180	104	7	468	4	171	347	1,E-14	70.9	DV703680.1
CC_TE-006	EC1	MuDRA	29.05	241	168	3	54	767	171	407	2,E-30	122	GT654814.1
CC_TE-007	SE4	MuDRA	26.80	291	209	7	9	869	265	522	3,E-22	95.5	DV698112.1
CC_TE-008	SE2	Activator_orf1	32.97	182	120	2	11	550	26	194	1,E-22	97.1	DV709520.1
CC_TE-009	SE2	Activator_orf1	32.42	182	121	2	6	545	26	194	2,E-22	96.7	DV709403.1
CC_TE-010	SE2	Activator_orf1	32.97	182	120	2	6	545	26	194	1,E-22	97.1	DV710276.1
CC_TE-011	SE4	Activator_orf1	34.69	147	95	2	43	480	50	194	3,E-21	92.4	DV699614.1
CC_TE-012	SH3	Activator_orf1	43.56	101	55	1	232	528	94	194	4,E-20	88.2	GT647332.1
CC_TE-013	SH3	Activator_orf1	43.56	101	55	1	231	527	94	194	3,E-20	88.2	GT647332.1
CC_TE-014	SE4	AtMu1	24.62	264	194	5	5	781	241	478	4,E-17	78.6	DV698112.1
CC_TE-015	SE4	AtMu1	25.00	184	138	3	184	735	302	459	3,E-12	55.8	DV699327.1
CC_TE-016	PP1	AtMu1	32.58	89	60	0	112	378	531	619	4,E-11	57.4	DV664869.1
CC_TE-017	SE3	AtMu1	25.16	159	104	1	112	543	536	694	3,E-11	58.9	DV679758.1
CC_TE-018	SE3	AtMu1	33.33	51	34	0	69	221	563	613	7,E-07	42.7	DV681403.1
CC_TE-019	SE3	AtMu1	33.33	51	34	0	30	182	563	613	2,E-06	42.7	DV681403.1
CC_TE-020	SE4	Jittery	34.76	233	151	2	70	765	164	394	1,E-39	152	DV694224.1
CC_TE-021	LF1	Jittery	41.18	119	70	0	193	549	226	344	3,E-27	100	DV693045.1
CC_TE-022	SE4	Jittery	45.37	205	111	1	17	628	227	431	2,E-57	188	DV698775.1
CC_TE-023	SE4	Jittery	43.24	259	146	1	20	793	227	485	4,E-61	224	DV698775.1
CC_TE-024	SE4	Jittery	37.60	242	150	2	16	738	262	495	3,E-40	155	DV703910.1
CC_TE-025	LF1	TAG2	31.23	269	180	6	75	866	275	522	1,E-27	107	DV693226.1
CC_TE-026	SE3	TAG2	35.03	197	127	2	18	605	218	413	2,E-33	132	DV687657.1
CC_TE-027	SE2	TAG1	32.14	224	151	4	951	283	492	705	9,E-36	140	DV705105.1
CC_TE-028	SE2	TAG1	30.16	252	175	4	1034	282	464	705	6,E-35	137	DV709915.1
CC_TE-029	SE4	Soymar	36.82	258	162	3	1253	483	167	422	2,E-37	145	DV703117.1

Table S2. Continuation.

**Retrotransposons**													
CC_TE-030	SE2	Del1	26.09	184	134	5	841	296	63	243	5,E-10	55.1	DV706511.1
CC_TE-031	SE2	Del1	50.00	154	75	4	5	460	983	1133	3,E-58	164	DV711314.1
CC_TE-032	PP1	Del1	26.79	168	122	4	602	102	60	222	5,E-10	54.7	DV678439.1
CC_TE-033	PP1	Del1	42.11	228	122	7	759	106	360	574	6,E-24	100	DV667846.1
CC_TE-034	PP1	Del1	38.75	160	97	5	578	102	376	525	2,E-22	94.7	DV666324.1
CC_TE-035	SE3	Del1	42.50	280	153	5	868	53	482	756	3,E-39	152	DV697852.1
CC_TE-036	SE3	Del1	42.05	264	145	4	828	61	494	756	5,E-42	160	DV697852.1
CC_TE-037	SE2	Del1	49.68	157	77	4	58	522	980	1133	5,E-68	165	DV711314.1
CC_TE-038	PP1	Del1	54.91	224	99	3	819	154	1201	1420	1,E-67	246	DV672748.1
CC_TE-039	PP1	Del1	50.92	163	78	3	632	150	1261	1420	3,E-58	164	DV675773.1
CC_TE-040	PP1	Del1	46.55	174	93	1	152	673	1268	1440	3,E-46	175	DV678143.1
CC_TE-041	PP1	Del1	55.86	145	62	2	584	156	1277	1420	2,E-42	161	DV664461.1
CC_TE-042	PP1	Del1	55.56	144	62	2	579	154	1278	1420	2,E-41	158	DV675773.1
CC_TE-043	PP1	Del1	55.63	142	61	2	574	155	1280	1420	3,E-41	157	DV675773.1
CC_TE-044	PP1	Del1	55.63	142	61	2	573	154	1280	1420	3,E-41	157	DV675773.1
CC_TE-045	PP1	Del1	56.03	141	60	2	571	155	1281	1420	4,E-41	157	DV675773.1
CC_TE-046	PP1	Del1	54.68	139	61	2	554	144	1283	1420	4,E-39	150	DV678515.1
CC_TE-047	PP1	Del1	55.97	134	57	2	549	154	1288	1420	6,E-39	149	DV673855.1
CC_TE-048	PP1	Dea1	75.32	158	39	0	131	604	2	159	5,E-72	248	DV665359.1
CC_TE-049	PP1	Dea1	56.50	177	75	3	129	653	2	176	3,E-45	171	DV678974.1
CC_TE-050	PP1	Dea1	75.24	105	26	0	102	416	23	127	1,E-37	145	DV675348.1
CC_TE-051	PP1	Dea1	67.16	67	22	0	246	446	61	127	3,E-37	102	DV674770.1
CC_TE-052	PP1	Dea1	37.04	270	168	2	906	103	70	339	1,E-43	166	DV665029.1
CC_TE-053	PP1	Dea1	64.20	162	58	0	616	131	74	235	1,E-59	218	DV675725.1
CC_TE-054	PP1	Dea1	70.37	108	32	1	429	106	94	200	9,E-41	156	DV676980.1
CC_TE-055	PP1	Dea1	43.02	172	95	1	619	113	103	274	3,E-37	144	DV664892.1
CC_TE-056	PP1	Dea1	44.16	154	83	1	560	108	121	274	5,E-33	130	DV664892.1
CC_TE-057	PP1	Dea1	44.37	151	81	1	551	108	124	274	6,E-33	129	DV664892.1
CC_TE-058	PP1	Dea1	58.52	229	95	0	784	98	131	359	2,E-73	265	DV678776.1
CC_TE-059	PP1	Dea1	55.97	134	59	1	175	576	137	265	2,E-37	146	DV675201.1
CC_TE-060	PP1	Dea1	41.57	166	97	0	605	108	139	304	4,E-36	140	DV676094.1
CC_TE-061	PP1	Dea1	69.80	245	74	0	845	111	158	402	1,E-100	355	DV671944.1

Table S2. Continuation.

CC_TE-062	PP1	Dea1	59.2	152	62	0	561	106	169	320	3,E-49	184	DV675233.1
CC_TE-063	PP1	Dea1	51.70	176	84	1	880	356	169	344	1,E-79	178	DV674709.1
CC_TE-064	PP1	Dea1	58.39	149	62	0	547	101	172	320	2,E-47	177	DV675233.1
CC_TE-065	PP1	Dea1	58.42	190	79	0	786	217	183	372	9,E-66	230	DV677919.1
CC_TE-066	PP1	Dea1	63.69	157	57	0	107	577	259	415	3,E-56	207	DV664560.1
CC_TE-067	PP1	Dea1	67.65	170	53	1	548	45	280	449	2,E-64	234	DV667983.1
CC_TE-068	PP1	Dea1	55.06	158	67	2	573	112	339	496	2,E-42	161	DV664378.1
CC_TE-069	PP1	Dea1	59.39	229	93	0	102	788	476	704	1,E-85	305	DV675167.1
CC_TE-070	PP1	Dea1	42.60	223	116	5	974	342	477	684	1,E-45	172	DV675167.1
CC_TE-071	PP1	Dea1	68.92	74	23	0	374	153	564	637	2,E-42	110	DV671895.1
CC_TE-072	PP1	Dea1	58.82	170	70	0	614	105	582	751	1,E-57	212	DV672803.1
CC_TE-073	PP1	Dea1	55.49	173	71	1	673	173	595	767	1,E-51	192	DV672796.1
CC_TE-074	PP1	Dea1	58.97	156	64	0	577	110	596	751	6,E-54	199	DV672803.1
CC_TE-075	PP1	Dea1	58.55	152	63	0	565	110	600	751	1,E-51	191	DV663349.1
CC_TE-076	PP1	Dea1	58.55	152	63	0	566	111	600	751	1,E-51	191	DV663349.1
CC_TE-077	PP1	Dea1	57.89	76	26	1	229	438	629	704	9,E-39	93.2	DV673511.1
CC_TE-078	PP1	Dea1	57.89	76	26	1	233	442	629	704	2,E-38	93.2	DV673511.1
CC_TE-079	PP1	Dea1	56.94	72	31	0	127	342	642	713	5,E-22	87.8	DV674975.1
CC_TE-080	PP1	Dea1	64.85	165	57	2	626	135	691	852	6,E-62	226	DV671957.1
CC_TE-081	PP1	Dea1	57.58	132	55	2	499	107	708	836	7,E-43	162	DV677003.1
CC_TE-082	PP1	Dea1	61.49	148	56	2	577	137	708	852	5,E-52	193	DV665761.1
CC_TE-083	PP1	Dea1	57.58	132	55	2	490	98	708	836	6,E-43	162	DV677003.1
CC_TE-084	PP1	Dea1	61.74	149	56	2	539	96	710	855	4,E-51	190	DV665288.1
CC_TE-085	PP1	Dea1	60.96	146	56	2	572	138	710	852	5,E-51	189	DV679089.1
CC_TE-086	SE2	Dea1	75.90	166	40	0	645	148	2	167	4,E-66	241	DV708559.1
CC_TE-087	SE2	Dea1	72.84	162	44	0	640	155	3	164	2,E-59	219	DV709537.1
CC_TE-088	SE2	Dea1	76.15	218	52	0	186	839	18	235	5,E-93	330	DV706425.1
CC_TE-089	SE2	Dea1	58.01	362	151	5	50	1132	66	421	1,E-114	402	DV710343.1
CC_TE-090	SE2	Dea1	60.67	328	129	1	53	1036	66	392	2,E-115	405	DV710343.1
CC_TE-091	SE2	Dea1	64.60	274	97	1	54	875	66	338	1,E-103	365	DV710343.1
CC_TE-092	SE2	Dea1	70.83	96	28	0	97	384	74	169	8,E-59	145	DV711728.1
CC_TE-093	SE2	Dea1	68.35	297	94	0	136	1026	92	388	1,E-122	422	DV710343.1
CC_TE-094	SE2	Dea1	67.54	191	61	1	252	821	124	314	1,E-80	266	DV710343.1
CC_TE-095	SE2	Dea1	67.91	134	43	0	554	153	267	400	2,E-49	186	DV709696.1
CC_TE-096	SE2	Dea1	45.24	42	23	1	1105	980	267	307	5,E-108	33.1	DV711786.1
CC_TE-097	SE2	Dea1	67.91	134	43	0	560	159	267	400	2,E-49	186	DV709696.1

Table S2. Continuation.

CC_TE-098	SE2	Dea1	67.91	134	43	0	557	156	267	400	2,E-49	186	DV709696.1
CC_TE-099	SE2	Dea1	67.91	134	43	0	554	153	267	400	2,E-49	186	DV709696.1
CC_TE-100	SE2	Dea1	57.79	353	141	3	1097	63	270	618	2,E-115	405	DV711786.1
CC_TE-101	SE2	Dea1	54.67	353	151	4	1087	56	270	618	3,E-103	364	DV711786.1
CC_TE-102	SE2	Dea1	58.36	353	139	3	1096	62	270	618	7,E-117	410	DV711786.1
CC_TE-103	SE2	Dea1	44.02	343	170	8	1097	135	271	594	1,E-59	220	DV711561.1
CC_TE-104	SE2	Dea1	56.90	348	142	4	1077	58	276	618	2,E-110	388	DV711786.1
CC_TE-105	SE2	Dea1	57.10	345	138	6	1064	60	280	618	7,E-106	373	DV711786.1
CC_TE-106	SE2	Dea1	56.56	343	141	3	1062	58	280	618	3,E-110	387	DV711786.1
CC_TE-107	SE2	Dea1	56.80	338	138	3	1050	61	285	618	1,E-107	379	DV711786.1
CC_TE-108	SE2	Dea1	58.63	336	131	3	1045	62	287	618	1,E-111	392	DV711786.1
CC_TE-109	SE2	Dea1	54.87	277	117	3	1037	231	288	560	1,E-103	290	DV711786.1
CC_TE-110	SE2	Dea1	57.53	332	133	3	1031	60	291	618	5,E-107	377	DV711786.1
CC_TE-111	SE2	Dea1	57.83	332	132	3	1028	57	291	618	9,E-111	380	DV711786.1
CC_TE-112	SE2	Dea1	58.13	332	130	4	1021	53	291	618	2,E-106	375	DV711786.1
CC_TE-113	SE2	Dea1	56.36	330	136	4	1023	58	294	618	7,E-103	363	DV711786.1
CC_TE-114	SE2	Dea1	58.66	329	128	3	1022	60	294	618	7,E-109	383	DV711786.1
CC_TE-115	SE2	Dea1	47.19	231	112	5	1006	344	298	524	2,E-70	163	DV713272.1
CC_TE-116	SE2	Dea1	58.81	318	123	3	987	58	305	618	8,E-108	370	DV711786.1
CC_TE-117	SE2	Dea1	58.54	316	123	3	982	59	307	618	3,E-104	368	DV711786.1
CC_TE-118	SE2	Dea1	58.86	316	122	3	981	58	307	618	5,E-108	370	DV711786.1
CC_TE-119	SE2	Dea1	58.58	309	120	3	963	61	314	618	3,E-101	358	DV711786.1
CC_TE-120	SE2	Dea1	59.15	306	117	3	949	56	317	618	1,E-101	359	DV711786.1
CC_TE-121	SE2	Dea1	59.67	305	115	3	949	59	318	618	1,E-104	361	DV708673.1
CC_TE-122	SE2	Dea1	34.98	243	153	4	937	224	323	564	1,E-35	140	DV711786.1
CC_TE-123	SE2	Dea1	60.00	300	112	3	932	57	323	618	5,E-103	358	DV706029.1
CC_TE-124	SE2	Dea1	60.00	300	112	3	932	57	323	618	2,E-101	358	DV704812.1
CC_TE-125	SE2	Dea1	47.41	251	122	5	325	1047	323	568	1,E-53	200	DV704812.1
CC_TE-126	SE2	Dea1	77.36	106	24	0	330	647	323	428	3,E-52	174	DV704812.1
CC_TE-127	SE2	Dea1	49.82	273	133	2	319	1125	323	595	6,E-72	260	DV711786.1
CC_TE-128	SE2	Dea1	59.33	300	114	3	934	59	323	618	2,E-99	352	DV711786.1
CC_TE-129	SE2	Dea1	59.67	300	113	3	935	60	323	618	5,E-101	357	DV664378.1
CC_TE-130	SE2	Dea1	58.66	283	109	3	888	64	340	618	8,E-97	332	DV711786.1
CC_TE-131	SE2	Dea1	58.16	282	110	3	877	56	341	618	1,E-99	328	DV711786.1
CC_TE-132	SE2	Dea1	49.17	240	114	3	756	61	382	617	1,E-66	222	DV704531.1

Table S2. Continuation.

CC_TE-133	SE2	Dea1	53.22	171	77	1	81	584	476	646	8,E-70	191	DV704200.1
CC_TE-134	SE2	Dea1	59.66	119	48	0	411	55	500	618	3,E-87	167	DV711786.1
CC_TE-135	SE2	Dea1	54.86	319	144	1	1	957	556	871	9,E-100	353	DV708284.1
CC_TE-136	SE2	Dea1	56.95	302	130	1	50	955	573	871	2,E-99	352	DV708284.1
CC_TE-137	SE2	Dea1	57.09	296	127	1	50	937	573	865	2,E-96	342	DV708284.1
CC_TE-138	SE2	Dea1	50.48	210	104	2	209	838	613	818	2,E-57	212	DV708127.1
CC_TE-139	SE2	Dea1	54.88	82	37	1	860	1105	724	803	2,E-31	89.0	DV710571.1
CC_TE-140	SE2	Dea1	60.90	312	116	3	137	1054	1	306	1,E-95	339	DV706425.1
CC_TE-141	SE1	Dea1	38.31	154	95	0	643	182	649	802	8,E-27	109	DV676590.1
CC_TE-142	PP1	Retrosat2	26.57	143	103	3	557	135	96	234	5,E-10	53.9	DV675703.1
CC_TE-143	PP1	Retrosat2	28.04	107	75	3	120	434	132	234	7,E-06	40.0	DV674841.1
CC_TE-144	PP1	Retrosat2	43.11	225	123	3	169	828	468	676	8,E-38	147	DV666206.1
CC_TE-145	PP1	Retrosat2	52.72	239	112	3	112	825	468	694	2,E-61	225	DV666442.1
CC_TE-146	PP1	Retrosat2	65.81	117	40	0	452	102	545	661	2,E-39	151	DV664893.1
CC_TE-147	PP1	Retrosat2	64.91	114	40	0	449	108	545	658	4,E-37	144	DV675566.1
CC_TE-148	PP1	Retrosat2	69.04	197	61	0	695	105	545	741	2,E-74	268	DV675887.1
CC_TE-149	PP1	Retrosat2	55.70	158	70	0	94	567	658	815	1,E-51	192	DV665338.1
CC_TE-150	PP1	Retrosat2	60.16	123	49	0	150	518	728	850	4,E-43	164	DV707537.1
CC_TE-151	PP1	Retrosat2	60.00	85	34	0	413	159	757	841	2,E-30	122	DV667306.2
CC_TE-152	PP1	Retrosat2	59.38	128	52	0	598	215	812	939	2,E-44	159	DV677919.2
CC_TE-153	PP1	Retrosat2	42.11	114	66	1	494	153	853	962	6,E-20	86.7	DV676893.1
CC_TE-154	PP1	Retrosat2	44.63	121	67	1	101	463	894	1013	2,E-22	95.1	DV675952.1
CC_TE-155	PP1	Retrosat2	66.32	95	32	0	392	108	1103	1197	7,E-38	147	DV665768.1
CC_TE-156	PP1	Retrosat2	44.88	254	138	4	905	150	1241	1488	4,E-56	207	DV672748.1
CC_TE-157	PP1	Retrosat2	36.11	108	69	1	492	169	1243	1344	3,E-15	70.9	DV679103.1
CC_TE-158	PP1	Retrosat2	54.90	153	68	1	549	94	1336	1488	5,E-43	163	DV675773.1
CC_TE-159	PP1	Retrosat2	54.90	153	68	1	610	155	1336	1488	6,E-43	163	DV675773.1
CC_TE-160	PP1	Retrosat2	54.90	153	68	1	605	150	1336	1488	6,E-43	163	DV675773.1
CC_TE-161	PP1	Retrosat2	53.03	132	61	1	544	152	1357	1488	3,E-40	136	DV672748.1
CC_TE-162	SE2	Retrosat2	66.54	269	90	0	65	871	545	813	2,E-103	365	DV706425.1
CC_TE-163	SE2	Retrosat2	55.18	299	134	5	67	963	545	831	1,E-82	296	DV706425.1
CC_TE-164	SE2	Retrosat2	65.16	221	77	0	716	54	546	766	8,E-81	290	DV708559.1
CC_TE-165	SE2	Retrosat2	64.25	221	79	0	714	52	546	766	5,E-79	284	DV708559.1
CC_TE-166	SE2	Retrosat2	65.16	221	77	0	716	54	546	766	7,E-81	290	DV708559.1

Table S2. Continuation.

CC_TE-167	SE2	Retrosat2	65.16	221	77	0	719	57	546	766	7,E-81	290	DV708559.1
CC_TE-168	SE2	Retrosat2	65.16	221	77	0	715	53	546	766	7,E-81	290	DV708559.1
CC_TE-169	SE2	Retrosat2	49.44	180	88	2	318	848	888	1065	5,E-50	173	DV704812.1
CC_TE-170	SE2	Retrosat2	46.95	213	110	2	315	944	888	1098	4,E-62	191	DV704812.1
CC_TE-171	SE2	Retrosat2	48.62	181	93	0	32	574	1034	1214	2,E-68	185	DV710255.1
CC_TE-172	SE2	Retrosat2	50.00	176	88	0	47	574	1039	1214	8,E-87	186	DV711314.1
CC_TE-173	SE2	Retrosat2	50.57	174	86	0	55	576	1041	1214	2,E-89	186	DV711314.1
CC_TE-174	SE2	Retrosat2	50.57	174	86	0	52	573	1041	1214	1,E-89	186	DV711314.1
CC_TE-175	SE2	Retrosat2	50.57	174	86	0	52	573	1041	1214	1,E-90	186	DV711314.1
CC_TE-176	SE2	Retrosat2	50.57	174	86	0	43	564	1041	1214	2,E-88	186	DV711314.1
CC_TE-177	SE2	Retrosat2	50.57	174	86	0	54	575	1041	1214	2,E-72	186	DV711314.1
CC_TE-178	SE2	Retrosat2	50.57	174	86	0	52	573	1041	1214	3,E-87	186	DV711314.1
CC_TE-179	SE2	Retrosat2	50.57	174	86	0	53	574	1041	1214	2,E-68	186	DV711314.1
CC_TE-180	SE2	Retrosat2	50.57	174	86	0	54	575	1041	1214	2,E-89	186	DV711314.1
CC_TE-181	SE2	Retrosat2	37.24	341	203	6	50	1039	1041	1366	9,E-60	220	DV711314.1
CC_TE-182	SE2	Retrosat2	50.88	171	84	0	62	574	1044	1214	2,E-88	185	DV711314.1
CC_TE-183	SE2	Retrosat2	50.88	171	84	0	59	571	1044	1214	2,E-60	185	DV709474.1
CC_TE-184	SE2	Retrosat2	50.88	171	84	0	64	576	1044	1214	3,E-85	185	DV711314.1
CC_TE-185	SE2	Retrosat2	50.88	171	84	0	63	575	1044	1214	8,E-90	185	DV712685.1
CC_TE-186	SE2	Retrosat2	50.88	171	84	0	65	577	1044	1214	6,E-94	185	DV711314.1
CC_TE-187	SE2	Retrosat2	51.19	168	82	0	84	587	1047	1214	3,E-71	190	DV708664.1
CC_TE-188	SE2	Retrosat2	50.90	167	82	0	74	574	1048	1214	8,E-90	182	DV711314.1
CC_TE-189	SE2	Retrosat2	49.13	173	87	1	80	595	1103	1275	1,E-44	169	DV704469.1
CC_TE-190	SE2	Retrosat2	49.13	173	87	1	84	599	1103	1275	2,E-44	169	DV704226.1
CC_TE-191	SE2	Retrosat2	49.13	173	87	1	81	596	1103	1275	2,E-44	169	DV704226.1
CC_TE-192	SE2	Retrosat2	49.13	173	87	1	80	595	1103	1275	2,E-44	169	DV704469.1
CC_TE-193	SE2	Retrosat2	49.69	163	81	1	111	596	1113	1275	4,E-43	165	DV704226.1
CC_TE-194	SE2	Retrosat2	49.35	154	77	1	139	597	1122	1275	5,E-40	154	DV704226.1
CC_TE-195	SE2	Retrosat2	49.13	173	87	1	83	598	1103	1275	1,E-44	169	DV704226.1
CC_TE-196	SE3	Retrosat2	39.85	133	79	1	493	98	686	818	2,E-31	88.6	DV682495.1
CC_TE-197	SE3	Retrosat2	54.81	104	46	1	764	456	852	955	6,E-48	119	DV680872.1
CC_TE-198	SE4	Cinful1_pol	48.15	135	70	0	459	55	271	405	2,E-34	135	DV699679.1
CC_TE-199	PP1	Cinful1_pol	39.83	118	71	0	452	99	282	399	4,E-25	104	DV674302.1
CC_TE-200	PP1	Cinful1_pol	37.82	119	74	0	456	100	282	400	3,E-24	102	DV674302.1
CC_TE-201	PP1	Cinful1_pol	35.27	224	141	2	764	105	780	1002	1,E-36	142	DV671835.1

Table S2. Continuation.

CC_TE-202	PP1	Cin4	38.06	134	82	2	511	113	512	642	1,E-19	86.7	DV667244.1
CC_TE-203	PP1	Cin4	38.06	134	82	2	511	113	512	642	1,E-19	86.7	DV667244.1
CC_TE-204	PP1	Cin4	38.06	134	82	2	510	112	512	642	1,E-19	86.7	DV667244.1
CC_TE-205	PP1	Cin4	38.06	134	82	2	509	111	512	642	1,E-19	86.7	DV667244.1
CC_TE-206	PP1	Cin4	33.94	109	65	3	523	218	803	907	2,E-14	69.7	DV674373.1
CC_TE-207	PP1	Opie2_pol	59.62	104	42	0	90	401	658	761	1,E-33	128	DV678310.1
CC_TE-208	PP1	Opie2_pol	57.71	201	85	0	709	107	799	999	5,E-63	230	DV668224.1
CC_TE-209	PP1	Opie2_pol	63.83	94	34	0	394	113	888	981	1,E-32	128	DV665543.1
CC_TE-210	PP1	Maggy_pol	41.53	183	104	1	644	105	319	501	6,E-47	150	DV674817.1
CC_TE-211	PP1	Maggy_pol	41.53	183	104	1	650	111	319	501	2,E-46	150	DV675132.1
CC_TE-212	PP1	Maggy_pol	41.53	183	104	1	650	111	319	501	5,E-47	150	DV675132.1
CC_TE-213	PP1	Osr1	41.38	232	133	4	109	795	290	519	1,E-47	173	DV665214.1
CC_TE-214	PP1	Osr1	42.41	224	126	4	111	773	290	511	4,E-45	171	DV665214.1
CC_TE-215	SE2	Osr1	58.77	114	47	0	534	193	1000	1113	1,E-53	139	DV708029.1
CC_TE-216	PP1	Retrosor1	45.19	104	57	0	414	103	929	1032	9,E-22	92.8	DV673217.1
CC_TE-217	SE2	Retrosor1	30.16	189	130	2	87	647	1595	1781	5,E-25	105	DV711831.1
CC_TE-218	SE4	AtRE2	46.32	285	152	1	60	911	885	1169	9,E-72	252	DV702240.1
CC_TE-219	SE4	AtRE2	46.32	285	152	1	62	913	885	1169	3,E-78	252	DV702240.1
CC_TE-220	PP1	TLC1_1	52.21	136	64	2	200	604	4	138	3,E-40	154	DV663970.1

Query id: arbitrary identification; Library: tissue, developmental stage or stress condition in which the clone was obtained (BP1 - Suspension cells treated with acibenzolar-S-methyl, CA1 - Non-embryogenic callus, CB1 - Suspension cells treated with acibenzolar-S-methyl and brassinosteroids, CL2 - Hypocotyls treated with acibenzolar-S-methyl, FB1 - Flower buds in stages 1 and 2—long, FR1 - Flower buds no 6, pinhead fruits no 1 and fruits (stages 1 and 2)—long, FR2 - Flower buds no 6, pinhead fruits no 1 and fruits (stages 1 and 2)—short, LV4 - Young leaves from orthotropic branch — long, LV5 - Young leaves from orthotropic branch—short, PA1 - Primary embryogenic callus, RT5 - Roots with acibenzolar-S-methyl, RT8 - Suspension cells stressed with aluminum, SH2 - Water deficit stresses plants (pool of tissues); **Subject id:** identification of the reference TEs (Table S3 in File S2).
