# Supplementary material for: Transcriptional Activity, Chromosomal Distribution and Expression Effects of Transposable Elements in Coffea Genomes
Source: PLoS One. 2013 Nov 11;8(11):e78931. doi: 10.1371/journal.pone.0078931 (PMC3823963; doi:10.1371/journal.pone.0078931)
Supplement: File S2 — Table S3. Completely characterized transposable elements library used for the classification into families of the expressed TEs identified in the Coffea transcriptome. (PDF) [file pone.0078931.s002.pdf]

FILE S2

Transcriptional activity, chromosomal distribution and expression effects of transposable elements in Coffea genomes

Fabrício R. Lopes[1,+], Daudi Jjingo[2,+], Carlos R. M. da Silva[3], Alan C. Andrade[4], Pierre Marraccini[4,5], João B. Teixeira[4], Marcelo F. Carazzolle[6], Gonçalo A. G. Pereira[6], Luiz Filipe P. Pereira[7], André L.L. Vanzela[3], Lu Wang[2], I. King Jordan[2,8] and Claudia M. A. Carareto*1

[1] Departamento de Biologia, UNESP , Univ. Estadual Paulista, São José do Rio Preto, SP, Brazil

[2]School of Biology, Georgia Institute of Technology, Atlanta, USA

[3] Departamento de Biologia Geral, Universidade Estadual de Londrina, Londrina, PR, Brazil

[4] EMBRAPA Recursos Genéticos e Biotecnologia (LGM), Brasília, DF, Brazil

[5]CIRAD, UMR AGAP, Montpellier, France

[6] Departamento de Genética, Evolução e Bioagentes, Universidade Estadual de Campinas, Campinas, SP, Brazil

[7]EMBRAPA Café, Brasília, Distrito Federal, Brazil

[8]PanAmerican Bioinformatics Institute, Santa Marta, Magdalena, Colombia

[+]These authors contributed equally to the work

*Corresponding author (phone: 55 17 3221 2382; fax 55 17 3221 2390; e-mail carareto@ibilce.unesp.br)

Table S3. Completely characterized transposable elements library used for the classification into families of the expressed TEs identified in the *Coffea* transcriptome. The description of this library includes both species and TE names, Genbank accession number (nt and aa) and References for the elements categorized in six groups (*Ty1/Copia*, *Ty3/Gypsy*, Non classified LTRs, *LINEs*, DNA Transposons, *MITEs*). Cells marked with "-" means non available sequences.

Species	TE name	Genbank accession number		Reference
		nucleotides	amino acids	
		Ty1/Copia		
Arabidopsis thaliana	Ta1-1	X53973	RTase: CAA37917	Konieczny et al., 1991
	Ta1-2	X53976	ORF1: CAA37923<br>ORF2: CAA37924<br>ORF3: CAA37925	Konieczny et al., 1991
	Ta1-3	X13291	CAA31653	Voytas and Ausubel, 1988
	AtRE1	AB021265	BAA78425	Kuwahara et al., 2000
	AtRE2	AB021264	BAA78424	Kuwahara et al., 2000
Hordeum vulgare	BARE-1	Z17327	Z17327	Manninen and Schulman, 1993
Lycopersicon peruvianum	Retrolyc1	AF228701	-	Costa et al., 1999
Nicotiana tabacum	Tnt1	X13777	CAA32025	Grandbastien et al., 1989
	Tto1	D83003	BAA11674	Hirochika et al., 1996
Oryza sativa	Osr1	-	BAB03249	Jwa, 2000
	Riccopia	M94492	AAA33902	Voytas et al., 1992.
Oryza longistaminata	Retrofit		AAB82754	Song et al., 1995
Saccharomyces cerevisiae	Ty1	-	Q07163	Boecke et al., 1998
Solanum tuberosum	Tst1	X52387	ORF1: CAA36613<br>ORF2: CAA36614<br>ORF3: CAA36615<br>ORF4: CAA36616	Camirand and Brisson, 1990
Solanum lycopersicon	TORTL1	U68072	-	Daraselia et al., 1996
Sorghum bicolor	pHind12	AF078902	-	Miller et al., 1998
Zea mays	PREM-2	U41000	AAB04689	Turcich et al., 1996
	Hopscotch	U12626	AAA57005	White et al., 1994
	Opie-2	U68408	gag: AAC49501<br>pol: AAC49502	SanMiguel et al., 1996
	Stonor4	AF082134	AAD12998	Marillonnet and Wessler, 1998
	Fourf	U68401 (LTR 5')	-	San Miguel et al., 1996.
	Victim	U68410 (LTR 5')	-	San Miguel et al., 1996.
	Ji-3	U68405 (LTR 5')	-	San Miguel et al., 1996.

Table S3. Continuation.

		Ty3/Gypsy		
Zea mays	Tekay	AF050455	-	SanMiguel and Bennetzen, 1998
	Cinful1	AF049110	pol: AAD11615	SanMiguel et al., 1996
	Cinful2	AF049111	-	SanMiguel et al., 1996
	Magellan	AF015269	-	Purugganan and Wessler, 1994
	Reina	U69258	-	Wing and Bennetzen, 1996
	Zeon1	U11059	gag: AAA93147	Hu et al., 1995
	Rle	AF057037	-	Wing and Bennetzen, 1996
Arabidopsis thaliana	Athila1	X81801	ORF1: CAA57397	Pelissier et al., 1995
Magnaporthe grisea	Maggy	L35053	gag: AAA33419<br>pol: AAA33420	Farman et al., 1996
	Pyret	AB062507	AB062507	Nakayashiki et al., 2001
Oryza sativa	RIRE1	D85597	BAA22288	Noma et al., 1987
	RIRE2	AB030283.1	gag: BAA84457<br>pol: BAA84458	Ohtsubo et al., 1999.
	RIRE3	AB014738	AB014738	Kumekawa et al., 1999
	Retrosat1	AF111709	gag/pol: AAD27548	Llaca et al., non published
	Retrosat2	AF111709	gag-pol: AAD27547	Llaca et al., non published
Sorghum bicolor	Retrosor1	AF098806	AAD19359	Llaca et al., non published
Lilium henryi	Del1-46	X13886	1510387A	Smyth et al., 1989
Ananas comosus	dea1	Y12432	CAA73042	Thomson et al., 1998
		**Non classified LTR**		
Arabidopsis thaliana	Endovir1-1	AY016208	AAG52950<br>gag/pol: AAG52949	Peterson-Burch et al., non published
	Frodo	AY923749	Not found	Kalendar and Schulman, non published
Brassica oleracea	Melmoth	Y12321	ORF1: CAA72989<br>ORF2: CAA72990	Pastuglia et al., non published
Lycopersicon esculentum	Jinling	DQ445619	Não encontrado	Wang et al. 2006.
	LARD1	DQ445621	Not found	Wang et al. 2006.
	LARD2	DQ445622	Not found	Wang et al. 2006.
Lycopersicon chilense	TLC1-1	AF279585	gag/pol: AAK29467	Tapia et al., 2005.
Solanum chacoenses	Sch2	U91993	-	Oosumi and Belknap, 1997
Solanum tuberosum	Potten1	U91987	-	Oosumi and Belknap, 1997
Zea mays	BS1	M25397	ORF1: AAA66269<br>ORF2: AAA66271<br>ORF3: AAA66270	Jin and Bennetzen, 1989

Table S3. Continuation.

		LINEs		
Arabidopsis thaliana	ATLINE1	AB016128	-	Noma et al., 1999.
	ATLINE2	AB016129	-	Noma et al., 1999.
	ATLINE3	AB016130	-	Noma et al., 1999.
	ATLINE4	AB016131	-	Noma et al., 1999.
Lilium speciosum	Del2	Z17425	-	Leeton and Smyth, 1993
Solanum tuberosum	POLN1	AB016140 (endonuclease)	-	Noma et al., 1999.
	POLN2	AB016141 (endonuclease)	-	Noma et al., 1999.
Solanum melongena	EGLN1	AB016142 (endonuclease)	-	Noma et al., 1999.
	EGLN2	AB016143 (endonuclease)	-	Noma et al., 1999.
	EGLN3	AB016144 (endonuclease)	-	Noma et al., 1999.
Zea mays	Cin4	Y00086	-	Schwarz-Sommer et al., 1987
		DNA Transposons		
Arabidopsis thaliana	AtMu1	AC012680.3	AAG52094	Singer et al., 2001
	AtMu2	AB023031.1	BAB 09991	Singer et al., 2001
	TAG1	L12220	AAC25101	Tsay et al., 1993
	TAG2	AF120335	AAD24567	Henk et al., 1999
	Lemi1	DQ888711	ABI75344	Loot et al., 2006.
	Limpet1	U76697	-	Klimyuk and Jones, 1997
	Harbinger	Repbase	-	Kapitonov and Jurka, 2005
	Sadhu	DQ385059	-	Ranghala et al., 2006.
Antirrhinum majus	Tam1	-	CAA40555	Nacken et al., 1991
	Tam2	X06266	-	Krebbers et al., 1987
	Tam3	X55078	CAA38906	Hehl et al., 1991
Glycine max	Soymar	AF078934	AAC28384	Jarvick and Lark, 1998
Ipomoea purpurea	Tip100	AB004906	BAA36225	Habu et al., 1998
Oryza sativa	Tnr3	D63711	-	Motohashi et al., 1996
Sorghum bicolor	Candystripe	AF206660	-	Chopra et al. 1999
Silene latifolia	Thelma13	-	AAP59878	Pritham et al., 2003
Zea mays	Activator	X01380	ORF1: CAA25635 ORF2: CAA25636 ORF3: CAA25637	Kunze and Starlinger, 1989
	BG	X56877	-	Hartings et al., non published
	Doppia4	AF187822	AAG17043	Bercury et al., 2001
	En-1	M25427	AAA66266	Pereira et al., 1986
	Jittery	AF247646	AAF66982	Xu et al., 2004
	MuDR	M76978	MuDRA: AAA21566 MuDRB: AAA21567	Hersberger et al., 1991
	Shooter	AF136220	AAD55677	Panavas et al., 1999

Table S3. Continuation.

		MITEs		
Arabidopsis thaliana	Emigrant	Z97337	Non-coding	Casacuberta et al., 1998
Oryza sativa	L-K	AF172282	Non-coding	Tarchini et al., 2000
	Wanderer	U34601	Non-coding	Ronald et al., 1992
	Explorer	U70541	Non-coding	Chen and Bennetzen, 1996
Zea mays	Tourist	S48688	Non-coding	Bureau and Wessler, 1992
	Heartbreaker	AF205927	Non-coding	Zhang et al., 2000
	Ditto	AC079830	Non-coding	Buel et al., non published
	Castaway	AC079830	Non-coding	Buel et al., non published
	Gaigin	AC079830	Non-coding	Buel et al., non published
	Stowaway	AC079830	Non-coding	Buel et al., non published
	Crackle	AC079830	Non-coding	Buel et al., non published
