# Supplementary material for: Transcriptional Activity, Chromosomal Distribution and Expression Effects of Transposable Elements in Coffea Genomes
Source: PLoS One. 2013 Nov 11;8(11):e78931. doi: 10.1371/journal.pone.0078931 (PMC3823963; doi:10.1371/journal.pone.0078931)
Supplement: File S3 — Table S4: List of fully cloned 27 cDNA from Coffea arabica similar to plant TEs. (PDF) [file pone.0078931.s003.pdf]

FILE S3

Transcriptional activity, chromosomal distribution and expression effects of transposable elements in *Coffea* genomes

Fabrício R. Lopes[1,+], Daudi Jjingo[2,+], Carlos R. M. da Silva[3], Alan C. Andrade[4], Pierre Marraccini[4,5], João B. Teixeira[4], Marcelo F. Carazzolle[6], Gonçalo A. G. Pereira[6], Luiz Filipe P. Pereira[7], André L.L. Vanzela[3], Lu Wang[2], I. King Jordan[2,8] and Claudia M. A. Carareto*1

[1] Departamento de Biologia, UNESP , Univ. Estadual Paulista, São José do Rio Preto, SP, Brazil

[2]School of Biology, Georgia Institute of Technology, Atlanta, USA

[3] Departamento de Biologia Geral, Universidade Estadual de Londrina, Londrina, PR, Brazil

[4] EMBRAPA Recursos Genéticos e Biotecnologia (LGM), Brasília, DF, Brazil

[5]CIRAD, UMR AGAP, Montpellier, France

[6] Departamento de Genética, Evolução e Bioagentes, Universidade Estadual de Campinas, Campinas, SP, Brazil

[7]EMBRAPA Café, Brasília, Distrito Federal, Brazil

[8]PanAmerican Bioinformatics Institute, Santa Marta, Magdalena, Colombia

[+]These authors contributed equally to the work

*Corresponding author (phone: 55 17 3221 2382; fax 55 17 3221 2390; e-mail carareto@ibilce.unesp.br)

Table S4. Percentage of amino acid similarity of 27 cDNA clones from *Coffea arabica* with plant TEs.

TE-derived cDNA			Comparison with Reference Elements (RE)				Comparison with Consensus Elements (CE)			
Name[1]	Library[2]	Size (bp)[3]	Description (protein length)[4]	SC/FS[5]	Similarity[6]	Aligned region (nt)[7]	Description[8]	SC/FS[5]	Similarity[6]	Aligned region (nt)[7]
DNA Transposons					48.5 ± 3.8				53 ± 3.2	
Ca_TE-009	FB1	1,481	*MuDRA* AAA21566 *Zm* (823 aa)	-/-	44	239/310-710/69	*MuDR4 Vv* (815 aa)	-/-	50	239/411-811/12
Ca_TE-008	LV5	1,799	“	-/-	44	239/178-710/6	“	-/-	51	239/300-811/6
Ca_TE-046	CB1	740	*AtMu1* AAG52094 *At* (761 aa)	-/-	41	254/556-699/114	*MuDR9 Vv* (679 aa)	-/-	44	269/514-673/15
Ca_TE-043	SH2	1,431	“	-/-	42	198/266-694/3	*MuDR4 Vv* (815 aa)	-/-	49	198/412-811/3
Ca_TE-044	LV4	869	“	-/-	35	171/451-693/8	“	-/-	42	159/593-814/17
Ca_TE-019	FB1	2,130	*Jittery* AAF66982 *Zm* (709 aa)	-/-	55	1,187/267-580/13	*MuDR-21 Vv* (769 aa)	-/-	55	1187/298-606/13
Ca_TE-035	CB1	1,227	*Tag2* AAD24567 *At* (577 aa)	-/-	49	257/252-542/55	*hAT-6 Vv* (652 aa)	-/-	53	245/329-648/7
Ca_TE-042	FR2	1,075	“	-/-	52	257/300-541/19	“	-/-	58	260/395-642/25
Ca_TE-050	SH2	1,402	*Tip100* BAA36225 *Ip (808* aa)	+/-	75	379/380-560/68	*hAT-4 Pt* (564 aa)	-/-	75	791/135-315/68
LTR Retrotransposons					62.9 ± 3.4				72.1 ± 2.3	
Ca_TE-061	SH2	773	*Retrosat2* AAD27547 *Os* (1,521 aa)	+/-	47	214/1344-1521/49	*Gypsy3 Pt* (1,407 aa)	+/-	68	256/1230-1397/34
Ca_TE-062	RT5	818	“	-/-	51	482/1351-1466/3	*Cop18-I Mt* (1,312 aa)	-/-	80	5/1175-1290/9
Ca_TE-063	FR2	1,078	*Cin4* Y00086 *Zm* (RTase 259 aa)	-/-	55	114/2-160/484	*Shaline14 Mt* (1,387 aa)	+/-	56	18/392-693/167
Ca_TE-064	RT8	1,582	“	-/-	58	1,072/2-162/24	“	-/-	55	127/179-659/30
Ca_TE-074	PA1	1,389	*Del1* 1510387A *Lh* (1443 aa)	-/-	73	1/5-175/875	*Gypsy2 Vv* (1,515 aa)	-/-	71	331/332-752/637
Ca_TE-080	RT5	363	*Dea1* CAA73042 *Ac* (871 aa)	-/-	68	113/613-692/10	*Gypsy3 Pt* (1,407 aa)	-/-	82	113/1068-1147/10
Ca_TE-072	CL2	552	*Tst1* CAA36615 *St* (675 aa)	+/+	60	5/58-203/292	*Tvv1 Vv* (1,382 aa)	-/+	75	2/319-556/46
Ca_TE-082	LV5	1,160	*Tst1* CAA36616 *St* (390 aa)	-/-	75	324/100-355/62	*Copia23 Vv* (318 aa)[9]	-/-	83	192/16-317/62
Ca_TE-085	CA1	941	*Tst1* CAA36614 *St* (334 aa)	-/-	49	293/52-260/15	*Copia35 Vv* (523 aa)[9]	-/+	68	1/88-314/360
Ca_TE-071	BP1	1,445	*Retrofit* AAB82754 *Ol* (1,445 aa)	-/-	37	65-513/11	*Copia32-I Vv* (1,803 aa)	-/-	74	1194-1674/5
Ca_TE-089	CL2	564	“	+/-	60	315/1348-1429/3	*Copia47 Pt* (564 aa)	+/-	84	249/468-569/3
Ca_TE-087	SH2	797	“	-/-	70	197/1233-1430/6	*Copia23 Vv* (318 aa)[9]	-/+	85	144/103-317/9
Ca_TE-088	CB1	747	*Hopscotch* AAA57005 *Zm* (1,439 aa)	-/-	70	307/1276-1416/17	*Copia23 Vv* (318 aa)[9]	-/-	74	247/159-317/23
Ca_TE-093	FR1	718	*Tnt1* CAA32025 *Nt* (1328 aa)	-/-	74	485/1277-1326/83	*Cop18-I Mt* (1,312 aa)	-/-	78	485/1261-1310/83
Ca_TE-094	CL2	362	*Ta1-1* CAA37917 *At* (587 aa)	-/-	76	8/447-563/3	*Gypsy-3 Pt* (1,407 aa)	+/-	68	5/923-1377/12
Ca_TE-095	CB1	1,284	*Osr1* BAB03249 *Os* (1,268 aa)	+/-	51	22/46-382/263	*Copia22 Pt* (316 aa)[9]	+/+	57	394/79-311/220
Ca_TE-096	LV4	854	*Athila1* CAA57397 *At* (935 aa)	-/-	59	249/52-248/14	*Gypsy1 Pt* (1,581 aa)	-/-	68	81/12-254/11
Ca_TE-098	CA1	1,067	*Ty1* Q07163 *Sc* (pol 1,756 aa)	-/+	100	2/494-846/5	-	-	-	-

cDNA similar to TEs from *C. arabica*: [1]Arbitrary identification (details see Material and Methods); [2]Tissue, developmental stage or stress condition in which the clone was obtained: BP1 - Suspension cells treated with acibenzolar-S-methyl, CA1 - Non-embryogenic callus, CB1 - Suspension cells treated with acibenzolar-S-methyl and brassinosteroids, CL2 - Hypocotyls treated with acibenzolar-S-methyl, FB1 - Flower buds in stages 1 and 2—long, FR1 - Flower buds no 6, pinhead fruits no 1 and fruits (stages 1 and 2)—long, FR2 - Flower buds no 6, pinhead fruits no 1 and fruits (stages 1 and 2)—short,

LV4 - Young leaves from orthotropic branch — long, LV5 - Young leaves from orthotropic branch—short, PA1 - Primary embryogenic callus, RT5 - Roots with acibenzolar-S-methyl, RT8 - Suspension cells stressed with aluminum, SH2 - Water deficit stresses plants (pool of tissues); [3]Size of the cDNA clone after full-length sequencing cloned into pSPORT1. Comparison with Reference Elements (completely characterized plant TEs): [4]TE name, Genbank accession number, species (At – *Arabidopsis thaliana*, Zm – *Zea mays*, Ac – *Ananas comosus*, Ol – *Oryza longistaminata*, Ip – *Ipoema purpurea*, Lh – *Lilium henryi*, Nt – *Nicotiana tabacum*, St – *Solanum tuberosum*, Sc – *Saccharomyces cerevisae*) and protein length; [5]Presence (+) or absence (-) of premature stop codon (SC) and frame shift (FR) in the TE´s sequence; [6]Percent similarity between putative *C. arabica* TE-encoded protein and reference element in BLASTx alignment; [7]Reference element amino acid region similar to our cDNA clone (central numbers), as well as nucleotides of the cDNA clone from *C. arabica* not included in the alignment in the both 5´ and 3´ ends (external numbers) as applied by Araujo *et al.* (2005). Comparison with Consensus Elements (stored in Repbase): [8]TE's family, species (Gm – *Glycine max*, Mt – *Medicago truncatula*, Pt – *Populus trichocarpa*, Vv – *Vitis vinifera*) and protein length, [9]Incomplete polyprotein.
