# Supplementary material for: Transcriptional Activity, Chromosomal Distribution and Expression Effects of Transposable Elements in Coffea Genomes
Source: PLoS One. 2013 Nov 11;8(11):e78931. doi: 10.1371/journal.pone.0078931 (PMC3823963; doi:10.1371/journal.pone.0078931)
Supplement: File S4 — Figures S1–S7. Supplementary Figures for the TE and gene expression analyses. (PDF) [file pone.0078931.s004.pdf]

File S4

Transcriptional activity, chromosomal distribution and expression effects of transposable elements in *Coffea* genomes

Fabrício R. Lopes[1,+], Daudi Jjingo[2,+], Carlos R. M. da Silva[3], Alan C. Andrade[4], Pierre Marraccini[4,5], João B. Teixeira[4], Marcelo F. Carazzolle[6], Gonçalo A. G. Pereira[6], Luiz Filipe P. Pereira[7], André L.L. Vanzela[3], Lu Wang[2], I. King Jordan[2,8] and Claudia M. A. Carareto*[1]

[1] Departamento de Biologia, UNESP , Univ. Estadual Paulista, São José do Rio Preto, SP, Brazil

[2]School of Biology, Georgia Institute of Technology, Atlanta, USA

[3] Departamento de Biologia Geral, Universidade Estadual de Londrina, Londrina, PR, Brazil

[4] EMBRAPA Recursos Genéticos e Biotecnologia (LGM), Brasília, DF, Brazil

[5]CIRAD, UMR AGAP, Montpellier, France

[6] Departamento de Genética, Evolução e Bioagentes, Universidade Estadual de Campinas, Campinas, SP, Brazil

[7]EMBRAPA Café, Brasília, Distrito Federal, Brazil

[8]PanAmerican Bioinformatics Institute, Santa Marta, Magdalena, Colombia

[+]These authors contributed equally to the work

*Corresponding author (phone: 55 17 3221 2382; fax 55 17 3221 2390; e-mail carareto@ibilce.unesp.br)

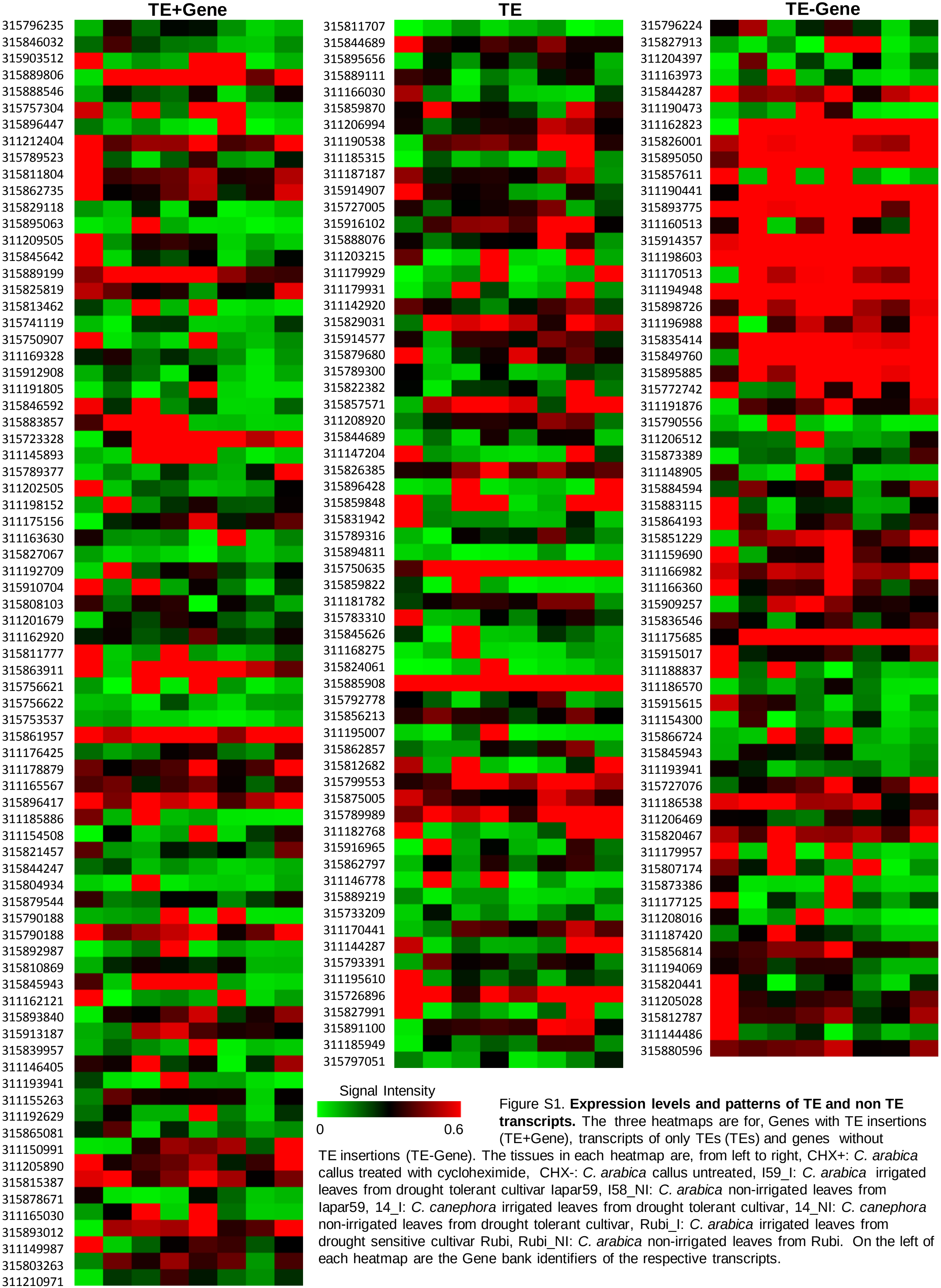

Figure S1. **Expression levels and patterns of TE and non TE transcripts.** The three heatmaps are for, Genes with TE insertions (TE+Gene), transcripts of only TEs (TEs) and genes without TE insertions (TE-Gene). The tissues in each heatmap are, from left to right, CHX+: *C. arabica* callus treated with cycloheximide, CHX-: *C. arabica* callus untreated, I59_I: *C. arabica* irrigated leaves from drought tolerant cultivar Iapar59, I58_NI: *C. arabica* non-irrigated leaves from Iapar59, 14_I: *C. canephora* irrigated leaves from drought tolerant cultivar, 14_NI: *C. canephora* non-irrigated leaves from drought tolerant cultivar, Rubi_I: *C. arabica* irrigated leaves from drought sensitive cultivar Rubi, Rubi_NI: *C. arabica* non-irrigated leaves from Rubi. On the left of each heatmap are the Gene bank identifiers of the respective transcripts.

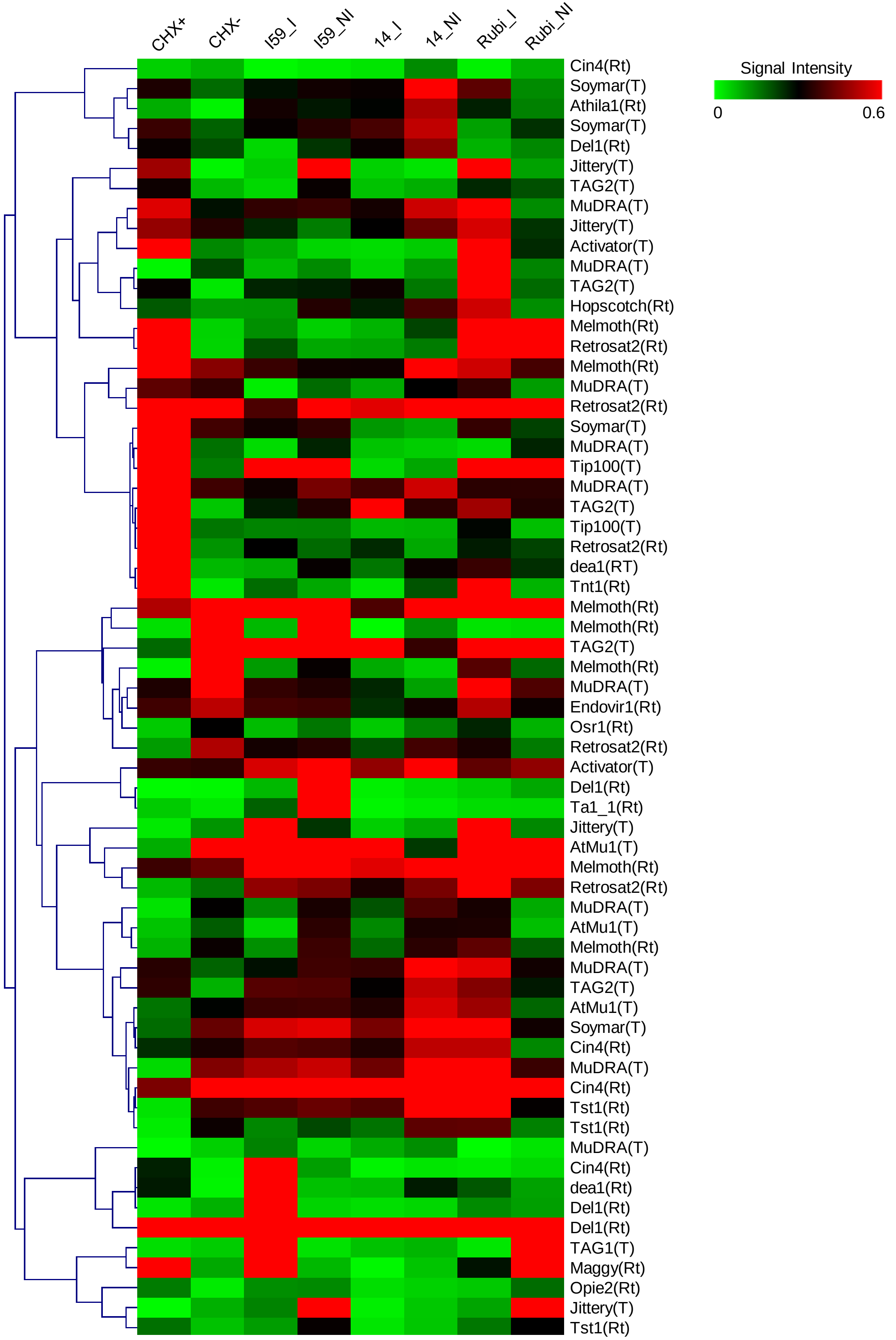

Figure S2. **Relationship between expression patterns and class of TE transcripts.** A hierarchical clustering of TE transcripts based on their expression patterns across eight tissues and conditions. TE classes are Rt (Retrotransposons) and T (DNA transposons). Tissues and conditions are CHX+: *C. arabica* callus treated with cycloheximide, CHX-: *C. arabica* callus untreated, I59_I: *C. arabica* irrigated leaves from drought tolerant cultivar Iapar59, I58_NI: *C. arabica* non-irrigated leaves from Iapar59, 14_I: *C. canephora* irrigated leaves from drought tolerant cultivar, 14_NI: *C. canephora* non-irrigated leaves from drought tolerant cultivar, Rubi_I: *C. arabica* irrigated leaves from drought sensitive cultivar Rubi, Rubi_NI: *C. arabica* non-irrigated leaves from Rubi.

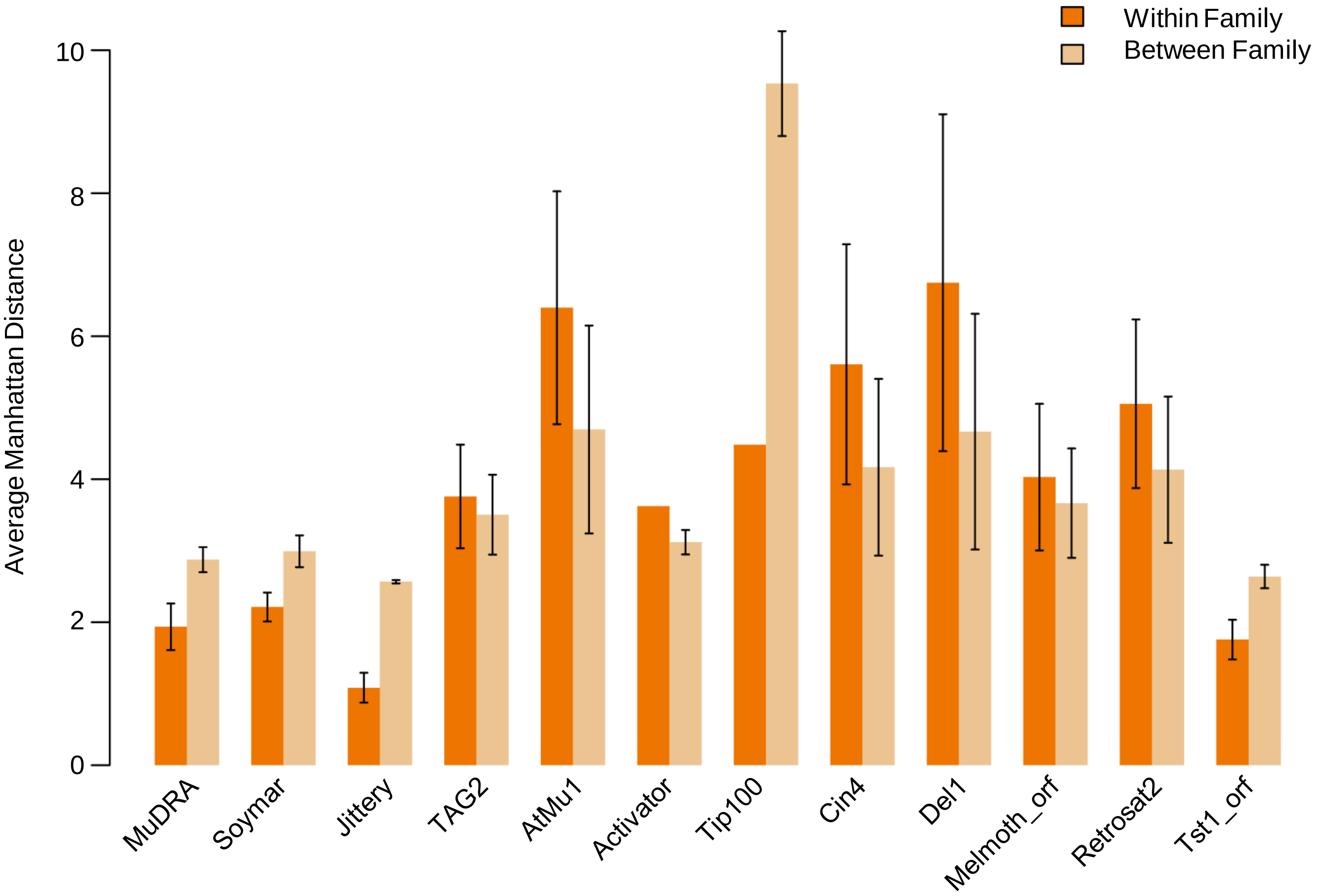

Figure S3. **Expression variation within and between TE families.** Intra and Inter TE family variation of expression as measured by average Manhattan distances.

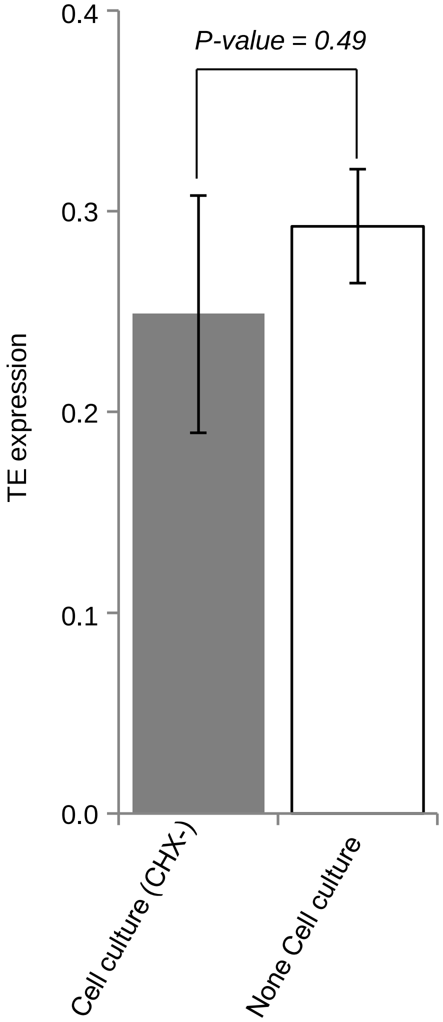

Figure S4. **Cell culture vs none-cell culture TE expression.** Average TE expression levels ± standard errors within cell-culture and none cell-culture tissues.

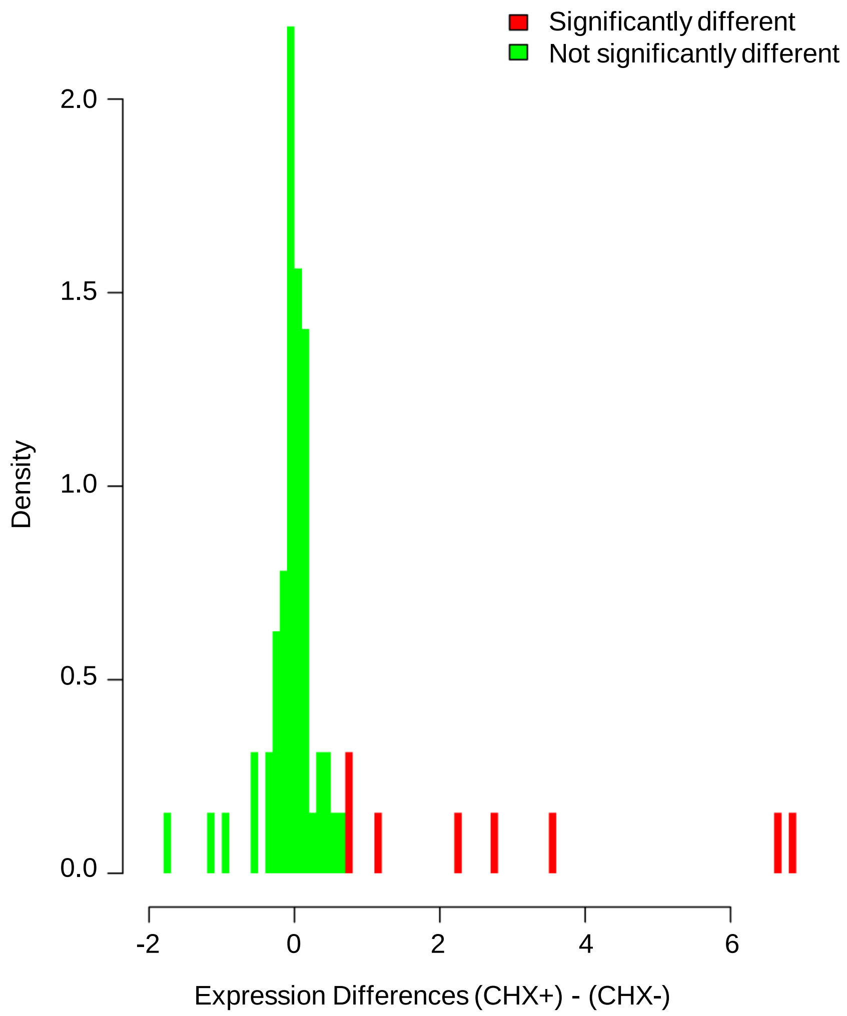

Figure S5. **Histogram of distributions of TE expression differences between CHX+ (*C. arabica* callus treated with cycloheximide) and CHX- (*C. arabica* callus untreated).** Values marked in red are significantly different from the average difference (z-test).

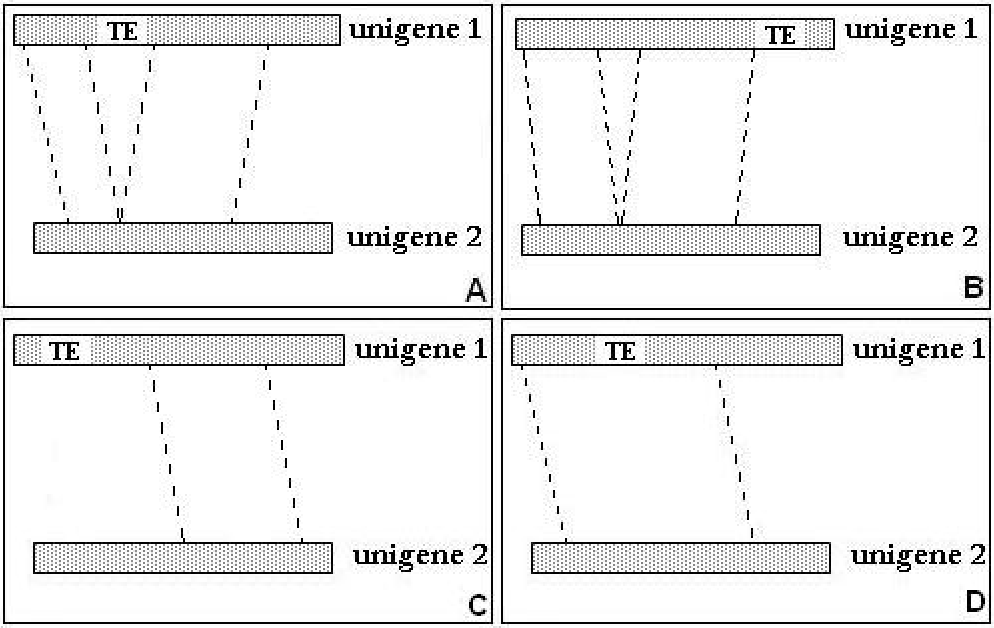

Figure S6. **Examples of alignments between unigenes containing TEs and the highly related unigenes using sequence similarity searches by BLASTn. (**A) Putative alternative splicing event. Non-conclusive splicing evidences. (B) The unigenes present one or more similarity regions, however, these regions finish before the start or begin after the TE insertion. (C) The similarity regions do not include the TE insertion site. (D) TE-cassette is within the similarity region between the unigenes. Hatched boxes represent the transcribed nucleotide sequence, the dashed lines represent the regions of similarity evaluated by BLASTn.

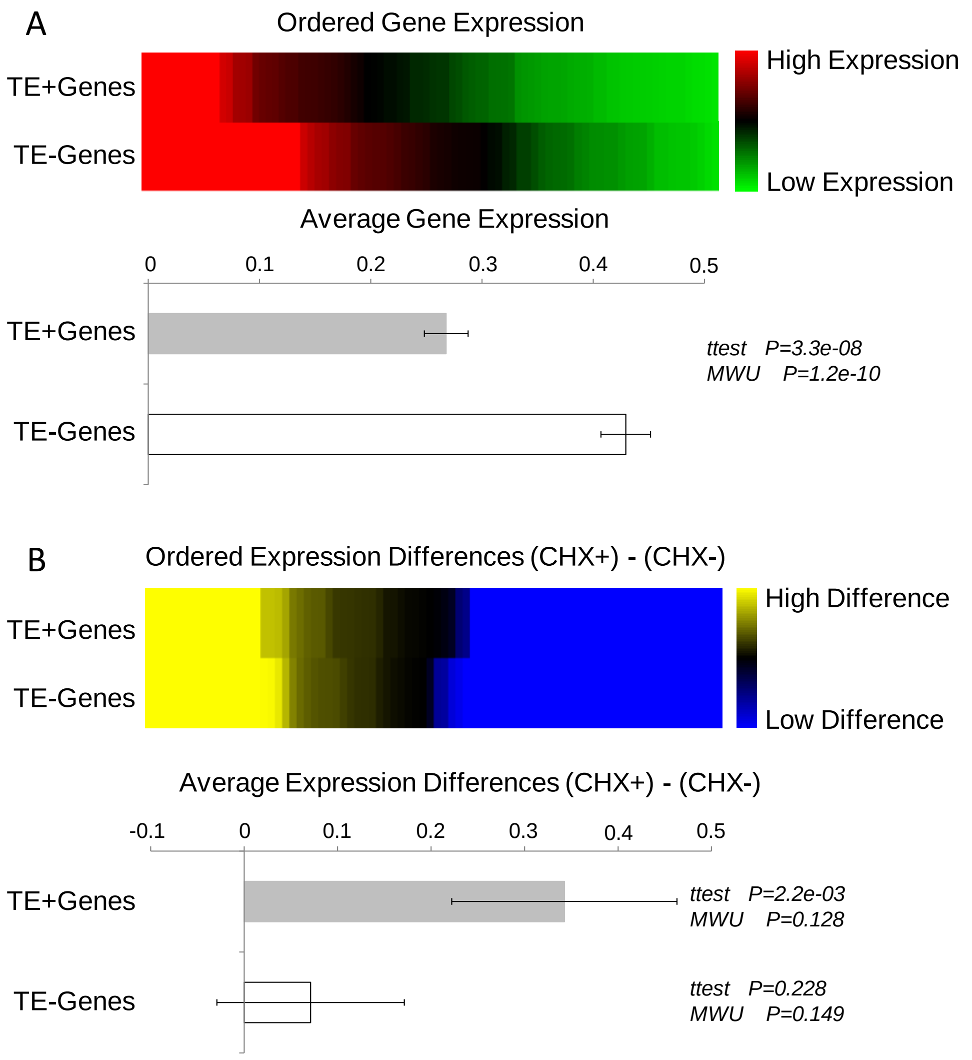

Figure S7. **Effect exonized TEs on gene expression.** (A) Comparison of overall expression levels of genes with TE cassettes (TE+Genes, *n=88*) versus genes with no TE cassettes (TE-Genes, *n=80*). Average expression levels ± standard errors were compared using the Students' t test and the Mann-Whitney U test (MWU) as indicated. (B) Differences in overall expression levels between CHX+ and CHX- conditions for TE+Genes versus TE-Genes. Average expression levels ± standard errors were compared between CHX+ and CHX- conditions for TE+Genes and TE-Genes individually using the Students' t test and the Mann-Whitney U test (MWU) as indicated.
