# Supplementary material for: Transcriptional Activity, Chromosomal Distribution and Expression Effects of Transposable Elements in Coffea Genomes
Source: PLoS One. 2013 Nov 11;8(11):e78931. doi: 10.1371/journal.pone.0078931 (PMC3823963; doi:10.1371/journal.pone.0078931)
Supplement: File S5 — Tables S5 & Table S6. List of unigenes containing or not TE–cassette insertions in C. arabica (Table S5) and C. canephora (Table S6). (PDF) [file pone.0078931.s005.pdf]

FILE S5

Transcriptional activity, chromosomal distribution and expression effects of transposable elements in Coffea genomes

Fabrício R. Lopes[1,+], Daudi Jjingo[2,+], Carlos R. M. da Silva[3], Alan C. Andrade[4], Pierre Marraccini[4,5], João B. Teixeira[4], Marcelo F. Carazzolle[6], Gonçalo A. G. Pereira[6], Luiz Filipe P. Pereira[7], André L.L. Vanzela[3], Lu Wang[2], I. King Jordan [2,8] and Claudia M. A. Carareto*1

[1] Departamento de Biologia, UNESP , Univ. Estadual Paulista, São José do Rio Preto, SP, Brazil

[2]School of Biology, Georgia Institute of Technology, Atlanta, USA

[3] Departamento de Biologia Geral, Universidade Estadual de Londrina, Londrina, PR, Brazil

[4] EMBRAPA Recursos Genéticos e Biotecnologia (LGM), Brasília, DF, Brazil

[5]CIRAD, UMR AGAP, Montpellier, France

[6] Departamento de Genética, Evolução e Bioagentes, Universidade Estadual de Campinas, Campinas, SP, Brazil

[7]EMBRAPA Café, Brasília, Distrito Federal, Brazil

[8]PanAmerican Bioinformatics Institute, Santa Marta, Magdalena, Colombia

[+]These authors contributed equally to the work

*Corresponding author (phone: 55 17 3221 2382; fax 55 17 3221 2390; e-mail carareto@ibilce.unesp.br)

Table S5. List of unigenes containing or not TE-cassette insertions in *C. arabica*.

unigenes containing TE-cassette insertions (“query”)			unigenes related those containing TE by BLASTn comparisons (“subject”)		
Identification	unigene length (TE insertion)	First protein hit in BLASTx searches (Genbank accession #)	Identification	unigene lenght (bp) [regions of similarity between “query”/”subject”]	First protein hit in BLASTx searches (Genbank accession #)
**unigenes identified by RepeatMasker**					
uni_CA_001	898 (585-641)	unknown protein (15230124)	uni_CA_032	1,039 [351-898/13-561]	unknown (15230124)
uni_CA_002	689 (502-623)	no hits found	uni_CA_060	724 [1-126/445-571]	no hits found
uni_CA_003	679 (192-280)	no hits found	uni_CA_061	671 [410-652/431-671]	no hits found
			uni_CA_062	718 [1-184/273-89]	unknown
			uni_CA_063	657 [425-574/219-369]	no hits found
			uni_CA_064	776 [1-216/166-381 ; 231-617/378-761]	unknown (21536582)
			uni_CA_065	705 [410-640/453-682]	no hits found
			uni_CA_066	593 [425-679/322-574]	no hits found
			uni_CA_067	915 [254-506/536-790]	no hits found
uni_CA_004	843 (386-461)	Calreticulin 1 precursor (11131631)	uni_CA_039	1,655 [1-329/696-1024 ; 331-586/1122-1380 ; 615-736/1534-1655]	Calreticulin 1 precursor (11131904)
			uni_CA_020	832 [26-326/6-308 ; 326-586/394-658]	Calreticulin 1 precursor (31249766)
			uni_CA_068	865 [123-202/2-81 ; 201-260/160-219 ; 258-329/304-375]	Calreticulin 1 precursor (11131631)
uni_CA_005	690 (62-243)	DRL1 (deformed roots and leaves 1) (23504265)	uni_CA_069	416 [1-162/35-198]	DRL1 (deformed roots and leaves 1) (15222972)
uni_CA_006	649 (14-103)	no hits found	uni_CA_049	1,014 [1-171/293-463 ; 173-584/603-1014]	no hits found
			uni_CA_070	1,087[1-171/366-736 ; 194-593/685-1080]	no hits found
Uni_CA_007	649 (164-219)	no hits found	uni_CA_049	1,014 [1-171/293-463 ; 173-584/603-1014]	no hits found
			uni_CA_070	1,087[1-171/366-736 ; 194-593/685-1080]	no hits found
uni_CA_008	763 (40-75)	probable kinesin heavy chain (7487566)	no hits found		
uni_CA_009	625 (141-231)	no hits found	no hits found		
uni_CA_010	454 (184-250)	no hits found	no hits found		
uni_CA_011	528 (64-234)	no hits found	uni_CA_013	618 [38-201/67-231 ; 113-495/229-613]	no hits found
			uni_CA_071	854[1-374/407-773]	Putative Cer1 (10716610)
			uni_CA_072	998 [1-202/796-998]	Putative Cer1 (37535278)
			uni_CA_073	2,116 [1-83/2034-2116]	Putative Cer1 (30678265)
uni_CA_012	822 (458-560)	unknown (15230463)	uni_CA_074	875 [67-609/78-621]	unknown (15230463)
uni_CA_013	618 (223-352)	no hits found	uni_CA_011	618 [67-231/38-201 ; 229-613/113-495]	no hits found
			uni_CA_071	854 [66-231/443-608 ; 229-492/519-773]	Putative Cer1 (10716610)
			uni_CA_072	998 [66-231/832-997 ; 229-319/908-998]	Putative Cer1 (37535278)
uni_CA_014	861 (458-560)	unknown (18401997)	no hits found		
uni_CA_015	651 (575-646)	unknown (15235843)	no hits found		
uni_CA_016	852 (379-483)	unknown (30678837)	no hits found		
uni_CA_017	751 (272-310)	protein kinase family - Ser/Thr protein kinase (15220275)	no hits found		

Table S5. Continuation

uni_CA_018	864 (624-669)	no hits found	no hits found		
uni_CA_019	685 (321-402)	no hits found	uni_CA_058	997 [18-647/369-997]	no hits found
			uni_CA_075	216 [449-2/663-216]	no hits found
uni_CA_020	832 (454-529)	putative calreticulin precursor (31249766)	uni_CA_039	1,655 [6-314/721-1027 ; 397-693/1120-1415]	calreticulin precursor (11131904)
			uni_CA_004	865 [6-308/26-326 ; 394-658/326-586]	calreticulin precursor (11131631)
			uni_CA_068	843 [102-184/1-81 ; 183-242/160-219 ; 240-397/304-461]	calreticulin precursor (11131631)
			uni_CA_076	316 [590-765/3-178]	no hits found
uni_CA_021	792 (603-644)	aldo/keto reductase family (15219786)	no hits found		
uni_CA_022	580 (81-159)	no hits found	uni_CA_023	721 [37-577/153-693]	no hits found
			uni_CA_077	808 [37-338/497-808]	no hits found
uni_CA_023	721 (197-275)	no hits found	uni_CA_022	580 [153- 693/37-577]	no hits found
			uni_CA_077	808 [1-454/345-808]	no hits found
uni_CA_024	836 (759-821)	unknown (15221754)	no hits found		
uni_CA_025	786 (603-770)	no hits found	no hits found		
uni_CA_026	871 (97-186)	no hits found	no hits found		
uni_CA_027	885(403-505)	no hits found	no hits found		
uni_CA_028	1,989 (809-892)	Probable MAP kinase phosphatase (13540262)	no hits found		
uni_CA_029	1,292 (504-542)	no hits found	uni_CA_078	891 [15-867/33-891]	no hits found
			uni_CA_079	654 [15-366/25-369 ; 836-1087/382-639]	no hits found
uni_CA_030	934 (29-85)	polyubiquitin 5 (70645)	uni_CA_080	1,544 [77-738/644-1305]	ubiquitin (902586)
			uni_CA_081	902 [66-771/38-742]	polyubiquitin (1332579)
			uni_CA_082	873 [51-692/47-688]	pentameric ubiquitin (602076)
			uni_CA_083	1,729 [66-692/57-683]	polyubiquitin (100934)
			uni_CA_084	793 [69-738/1-670]	ubiquitin (902586)
			uni_CA_085	1,342 [79-774/532-1227]	ubiquitin (70644)
			uni_CA_086	857 [127-774/1-648]	hexameric polyub. (170352)
			uni_CA_087	886 [69-681/1-615]	polyubiquitin (30680052)
			uni_CA_088	1,097 [127-774/335-981]	hexameric polyubiquitin (170352)
			uni_CA_089	1,540 [79-762/579-1262]	ubiquitin (4115337)
			uni_CA_090	695 [77-440/275-640]	polyubiquitin (1332579)
			uni_CA_091	1,315 [77-740/530-1193]	polyubiquitin (3126967)
			uni_CA_092	960 [75-737/86-748]	polyubiquitin (1332579)
			uni_CA_093	1,575 [75-737/846-1507]	polyubiquitin (1332579)
			uni_CA_094	793 [148-771/603-1226]	polyubiquitin (1332579)
			uni_CA_095	1,410 [75-680/62-668]	polyubiquitin (70645)
			uni_CA_096	1,482 [75-737/96-758]	polyubiquitin (481477)
			uni_CA_097	762 [66-738/46-717]	polyubiquitin (421929)
			uni_CA_098	822 [136-755/123-740]	polyubiquitin (1332579)
uni_CA_031	762 (379-480)	fertility restorer (22128587)	No hits found		
uni_CA_032	1,039 (247-303)	6b-interacting protein 1 (15230124)	uni_CA_001	898 [13-561/351-898]	unknown (15230124)
uni_CA_033	1,118 (997-1106)	transfactor-like (30699418)	No hits found		

Table S5. Continuation

uni_CA_034	1,135 (146-233)	heavy-metal-associated domain-containing protein (42572261)	uni_CA_099	501 [702-1135/1-434]	same (30679432)
			uni_CA_100	339 [1023-1135/52-164]	no hits found
uni_CA_035	1.093	unknown (42569311)	uni_CA_101	859 [1-713/148-859]	unknown (26452742)
			uni_CA_102	896 [1-650/58-706]	unknown (30678837)
uni_CA_036	1,990 (1462-1590)	rab GDP dissociation inhibitor (7489133)	uni_CA_103	307 [58-665/50-656]	No hits found
uni_CA_037	1,316 (185-244)	PSTVd RNA-binding protein Virp1a (10179602)	uni_CA_104	845 [298-1080/1-784]	no hits found
			uni_CA_051	2,655 [53-264/1740-1951 ; 360-503/1951-2094 ; 590-1084/2093-2588]	same
			uni_CA_105	367 [946-1312/1-367]	no hits found
			uni_CA_106	813 [53-184/448-579]	viroid RNA-binding protein (19171209)
uni_CA_038	2,054 (2-61)	glyceraldehyde-3-phosphate dehydrogenase, cytosolic (18072805)	uni_CA_107	1,298 [46-364/359-680 ; 373-978/690-1298]	same
			uni_CA_108	1,139 [1013-1158/1-146 ; 1155-1436/356-637 ; 1467-1937/660-1132]	same
			uni_CA_109	721 [1314-1436/17-139 ; 1432-1554/286-406]	same
			uni_CA_110	650 [46-128/558-650]	no hits found
uni_CA_039	1,655 (1177-1252)	calreticulin precursor (11131904)	uni_CA_111	876 [146-711/304-865]	calreticulin 2 (15217459)
			uni_CA_020	832 [721-1027/6-314]	calreticulin percusor (31249466)
			uni_CA_004	843 [696-1024/1-329 ; 1122-1380/331-586 ; 1534-1655/615-736]	calreticulin percusor (11131631)
			uni_CA_076	379 [1311-1461/1-149]	no hits found
			uni_CA_112	823 [1460-658-809]	molecular chaperone Hsp90-1 (38154489)
			uni_CA_068	865 [817-897/1-81 ; 953-1027/304-378 ; 1021-1120/458-557]	calreticulin percusor (11131631)
			uni_CA_113	162 [1528-1632/54-158]	no hits found
uni_CA_040	2,286 (750-817)	multidomain cyclophilin type peptidyl-prolyl cis-trans isomerase - CYP63 (15229425)	uni_CA_114	860 [1446-1695/62-311]	same
uni_CA_041	961 (87-142)	SRG1 (senescence-related gene 1), oxidoreductase, 2OG-Fe(II) oxygenase family (15219988)	uni_CA_115	936 [1-897/44-936]	same
			uni_CA_116	2,265 [139-961/1-824]	same
			uni_CA_117	900 [162-934/1-773]	same
			uni_CA_118	773 [380-961/1-583]	Same
			uni_CA_119	691 [641-961/1-322]	SRG1 homolog (25285681)
			uni_CA_120	677 [737-961/1-224]	SRG1 like protein (26451337)
			uni_CA_121	482 [870-961/3-95]	SRG1 like protein (26451337)
uni_CA_042	1,102 (46-118)	ribosomal protein L7 (445613)	uni_CA_122	765 [102-803/60-765]	same
			uni_CA_123	877 [102-280/107-285 ; 278-809/347-877]	same
			uni_CA_124	1,143 [101-838/219-956]	same
uni_CA_043	1,998 (349-447)	ubiquitinating enzyme (49328017)	No hits found		
uni_CA_044	656 (382-445)	no hits found	No hits found		
uni_CA_045	909 (373-451)	no hits found	No hits found		
uni_CA_046	861 (415-495)	rust resistance Rp1-D-like protein (13310480)	uni_CA_125	911 [307-422/1-117 ; 503-856/135-488]	similar to NBS-LRR type resistance protein (28564572)
			uni_CA_126	701 [503-861/59-416]	putative NBS-LRR type resistance protein (13872974)
			uni_CA_127	820 [112-416/91-396 ; 497-861/411-774]	putative disease resistant protein rga4 (32470648)
			uni_CA_128	874 [122-352/295-526]	Vrga1 (6606266)

Table S5. Continuation.

uni_CA_047	1,075 (433-512)	pre-mRNA splicing factor cwc15/Cwc15 cell cycle control (21592359)	No hits found		
uni_CA_048	714 (480-527)	CONSTANS-like protein (18390719)	uni_CA_129	890 [27-714/3-681]	same
uni_CA_049	1,014 (282-366)	no hits found	uni_CA_070	1,081 [1-535/74-608 ; 531-625/616-710 ; 624-685/1014-1071]	no hits found
			uni_CA_007	649 [293-463/1-171 ; 603-173/1014-584]	no hits found
uni_CA_050	1,729 (1323-1372)	sucrose synthase (29289943)	uni_CA_130	660 [731-1391/4-660]	same
			uni_CA_131	259 [1489-1729/23-252]	no hits found
			uni_CA_132	286 [1585-1729/135-274]	no hits found
uni_CA_051	2,655 (1872-1931)	PSTVd RNA-binding protein Virp1a (10179602)	uni_CA_133	881 [113-419/9-315 ; 1043-1604/320-881]	same
			uni_CA_104	845 [1951-2094/63-206 ; 2093-2635/293-834]	no hits found
			uni_CA_037	1,316 [2093-2588/590-1084]	same
			uni_CA_106	813 [1651-1871/358-579]	same
			uni_CA_134	872 [570-1149/55-635]	same
			uni_CA_105	367 [2449-2588/1-139]	no hits found
uni_CA_052	759 (520-590)	no hits found	uni_CA_135	870 [13-244/591-818]	no hits found
uni_CA_053	1,274 (39-109)	transmembrane MLO family protein (18398444)	uni_CA_136	764 [920-1234/14-354]	same
			uni_CA_137	872 [928-1234/48-354]	transmembrane MLO protein (201398)
uni_CA_054	1,017 (606-685)	unknown (30681632)	No hits found		
uni_CA_055	1,208 (185-244)	universal stress protein (USP) family protein (30681955)	uni_CA_138	1,166 [38-101/5-68 ; 277-607/63-393 ; 606-701/477-572 ; 702-1140/663-1105]	same
uni_CA_056	750 (469-550)	Unknown protein (18413696)	No hits found		
uni_CA_057	439 (119-201)	Unknown protein (18417290)	No hits found		
uni_CA_058	997 (672-754)	no hits found	uni_CA_019	685 [369-997/18-647]	no hits found
			uni_CA_075	216 [797-994/2-197]	no hits found
uni_CA_059	1,185 (798-876)	nucleosome assembly protein (NAP) (15221298)	uni_CA_139	676 [10-375/33-398 ; 561-839/398-676]	nucleosome/chromatin assembly factor A (46805241)
**unigenes identified by tBLASTx**					
uni_CA_140	786 (527-694)	galactokinase GHMP kinase-like (2326372)	No hits found		
uni_CA_141	829 (649-798)	GHMP kinase-like protein (42409310)	No hits found		
uni_CA_142	856 (429-575)	SC35-like putative splicing factor (31249706)	uni_CA_145	798 (337-427/1-91 ; 427-705/205-483 ; 714-856/480-623)	same
uni_CA_143	781 (485-631)	SC35-like putative splicing factor (31249706)	uni_CA_146	1613 (1-192/163-354 ; 404-778/350-721)	SC35-like splicing factor (9843661)
			uni_CA_147	1177 (1-192/119-310 ; 404-778/306-677)	same
			uni_CA_142	856 (418-479/441-502)	same
			uni_CA_145	798 (418-479/219-280)	same
uni_CA_144	662 (254-409)	SC35-like putative splicing factor (31249706)	uni_CA_167	760 (124-497/188-566)	SC35-like splicing factor (30687014)
			uni_CA_148	649 (192-269/546-623)	putative pre-mRNA splicing factor (47497118)

Table S5. Continuation.

uni_CA_145	798 (201-353)	SC35-like putative splicing factor (31249706)	uni_CA_142	856 (1-91/337-427 ; 205-483/427-705 ; 480-623/714-856)	same
			uni_CA_143	781 (219-280/418-479)	same
			uni_CA_146	1613 (219-280/364-425)	SC35-like splicing factor (9843661)
			uni_CA_147	1177 (219-286/320-387)	Same
uni_CA_146	1,613 (352-498)	SC35-like putative splicing factor	uni_CA_147	1177 (59-957/15-913 ; 954-1201/929-1177 ; 1170-1292/1005-1129)	same
			uni_CA_143	781 (163-354/1-192 ; 350-721/404-778)	same
			uni_CA_142	856 (364-425/441-502)	same
			uni_CA_145	798 (364-425/219-280)	same
uni_CA_147	1,177 (308-454)	SC35-like putative splicing factor (31249706)	uni_CA_146	1613 (15-913/59-957 ; 929-1177/954-1201 ; 1005-1129/1170-1292)	SC35-like splicing factor (9843661)
			uni_CA_143	781 (306-677/404-778 ; 119-310/1-192)	same
			uni_CA_142	856 (320-387/441-508)	same
			uni_CA_145	798 (320-387/219-286)	same
uni_CA_148	1,195 (608-763)	SC35-like putative splicing factor (47497118)	uni_CA_168	768 (13-692/86-795)	unknown (12002297) - SC35
			uni_CA_169	756 (13-263/86-336 ; 235-361/341-467)	no hits found
			uni_CA_144	662 (546-623/192-269)	SC35-like splicing factor (30687014)
uni_CA_149	847 (414-824)	protein F21D18.16 (25405918)	No hits found		
uni_CA_150	951 (303-500)	heat shock cognate 70 kd protein (123650)	uni_CA_153	2475 (40-951/141-1053)	heat shock cognate protein 70 (26985223)
			uni_CA_154	2209 (87-951/51-916)	heat shock cognate protein 70 (26985221)
			uni_CA_152	901 (87-817/151-877)	heat shock cognate protein 70 (26985221)
			uni_CA_151	2340 (87-951/122-987)	dnaK-type molecular chaperone hsp70 (1076746)
			uni_CA_170	768 (86-280/342-536)	heat shock cognate protein 70 (26985219)
			uni_CA_171	751 (189-316/247-374 ; 363-490/421-548)	heat shock protein (6969976)
			uni_CA_172	768 (184-280/199-295)	molecular chaperone HSP71.2 (1076529)
uni_CA_151	2,340 (338-535)	dnaK-type molecular chaperone hsp70 (1076746)	uni_CA_153	2475 (122-1974/188-2040)	heat shock cognate protein 70 (26985223)
			uni_CA_154	2209 (114-1825/43-1754 ; 1907-1977/1836-1906)	heat shock cognate protein 70 (26985221)
			uni_CA_173	722 (1353-1452/1-100 ; 1415-1439/122-98 ; 1429-1993/116-683)	Same
			uni_CA_174	402 (1892-2147/1-248 ; 2173-2306/269-402)	heat shock protein (6969976)
			uni_CA_150	951 (122-987/87-951)	heat shock cognate 70 kd protein (123650)
			uni_CA_152	901 (114-816/143-843)	heat shock cognate protein 70 (26985221)
			uni_CA_170	768 (122-449/343-670)	Same
			uni_CA_175	804 (880-1284/12-415)	molecular chaperone Nthsp70 (100335)
			uni_CA_176	900 (1289-1506/81-297 ; 1602-1857/340-598)	heat shock protein hsp70 (15230534)
			uni_CA_177	804 (947-1026/386-465 ; 1112-1259/549-696)	heat shock protein (6969976)
			uni_CA_178	751 (572-678/595-701)	heat shock cognate 70 kd protein (123650)
			uni_CA_179	1,888 (546-639/56-149)	Luminal binding protein 5 precursor (729623)
			uni_CA_171	969 (546-639/650-743)	Luminal binding protein 5 precursor (729623)
			uni_CA_172	768 (221-300/201-280 ; 554-619/534-599)	heat shock protein hsp70 (15230534)
			uni_CA_180	824 (569-625/220-276)	Luminal binding protein precursor (1346172)
			uni_CA_181	2,591 (569-625/825-881)	heat shock protein (6969976)
				1,238 (575-639/10-74)	Luminal binding protein 5 precursor (729623)

Table S5. Continuation.

uni_CA_152	901 (367-564)	non-cell-autonomous heat shock	uni_CA_154	2,209 (101-901/1-806)	same
		cognate protein 70 (26985221)	uni_CA_153	2475 (151-878/188-918)	heat shock cognate protein 70 (26985223)
					same
			uni_CA_150	951 (151-877/87-817)	dnaK-type molecular chaperone hsp70
			uni_CA_151	2,340 (143-843/114-816)	(1076746)
					heat shock protein (6969976)
			uni_CA_177	804 (217-380/409-572)	
uni_CA_153	2,475 (404-601)	cell-autonomous heat shock cognate	uni_CA_150	951 (143-1053/42-951)	heat shock cognate protein 70 (26985221)
		protein 70 (26985223)	uni_CA_154	2,209 (188-1861/51-1724 ; 1899-2045/1762-1908)	heat shock cognate protein 70 (26985221)
			uni_CA_151	2,340 (188-2040/122-1974)	dnaK-type molecular chaperone hsp70
					(1076746)
			uni_CA_152	901 (188-918/151-878)	heat shock cognate protein 70 (26985221)
			uni_CA_173	722 (1419-1518/1-100 ; 1508-1852/129-477)	same
			uni_CA_175	804 (983-1335/48-400)	molecular chaperone Nthsp70 (100335)
			uni_CA_176	900 (1358-1584/84-309 ; 1670-1861/342-533 ; 1943-	heat shock protein hsp70 (15230534)
				2022/615-694)	
			uni_CA_183	711 (1821-1853/69-101 ; 1868-2095/116-344)	heat shock protein 70 (21327033)
			uni_CA_177	804 (939-1059/311-432 ; 1178-1325/549-696)	heat shock protein (6969976)
			uni_CA_171	721 (290-417/247-374 ; 464-567/421-524)	heat shock cognate 70 kd protein (123650)
			uni_CA_170	768 (187-510/342-665)	molecular chaperone Nthsp70 (100335)
			uni_CA_174	402 (1958-2040/1-83)	heat shock cognate 70 kd protein (123650)
uni_CA_154	2,209 (267-464)	non-cell-autonomous heat shock	uni_CA_152	901 (1-806/101-901)	same
		cognate protein 70 (26985221)	uni_CA_153	2,475 (51-1724/188-1861 ; 1762-1908/1899-2045)	heat shock cognate protein 70 (26985223)
					dnaK-type molecular chaperone hsp70
			uni_CA_151	2,340 (43-1754/114-1825 ; 1836-1906/1907-1977)	(1076746)
					heat shock protein hsp70 (15230534)
			uni_CA_176	900 (1217-1472/80-334 ; 1523-1975/332-790)	heat shock protein (6969976)
			uni_CA_177	804 (555-1269/64-777)	heat shock cognate 70 kd protein (123650)
			uni_CA_150	951 (51-916/87-956)	No hits found
					molecular chaperone Nthsp70 (100335)
			uni_CA_184	277 (1976-2140/110-274)	dnaK-type molecular chaperone hsp70
			uni_CA_175	804 (846-1208/48-410 ; 1281-1381/483-583)	(1076746)
			uni_CA_173	722 (138-271/189-322)	molecular chaperone Nthsp70 (100335)
					chaperone HSP71.2 (1076529)
			uni_CA_170	768 (117-301/409-593)	heat shock cognate 70 kd protein (123650)
			uni_CA_172	768 (138-271/189-322)	heat shock cognate 70 kd protein (123650)
			uni_CA_171	751 (133-271/227-365)	Luminal binding protein 5 precursor
					(729623)
			uni_CA_174	402 (1836-1906/16-86)	Luminal binding protein 5 precursor
					(729623)
			uni_CA_178	1,888 (475-559/56-140)	
			uni_CA_179	969 (475-650/559-734)	
uni_CA_155	884 (12-482)	EIL3 (14280044)	uni_CA_185	840 (40-190/674-820)	EIN3-like protein (15425735)
			uni_CA_157	2,580 (63-370/875-1182)	EIL2 (30016896)
uni_CA_156	1123	EIL3 (30016898)	uni_CA_157	2,580 (298-423/13-142 ; 421-1123/205-907)	EIL2 (30016896)
			uni_CA_186	1,297 (735-926/12-203)	EIL2 (30016896)
			uni_CA_187	753 (650-840/96-294)	EIL2 (14280042)
			uni_CA_188	806 (644-704/97-157 ; 791-918/247-374)	EIN3-like protein (15425735)
			uni_CA_185	840 (644-704/247-307 ; 791-918/397-524)	EIN3-like protein (15425735)

Table S5. Continuation.

uni_CA_157	2,580 (827-1294)	EIL2 (30016896)	uni_CA_186	1,297 (519-710/12-203 ; 1405-2481/195-1270)	EIL2 (30016896)
			uni_CA_156	1,123 (13-142/298-423 ; 205-907/421-1123)	EIL3 (30016898)
			uni_CA_155	884 (875-1182/63-370)	EIL3 (14280044)
			uni_CA_188	806 (428-488/97-157 ; 575-702/247-374)	EIN3-like protein (15425735)
			uni_CA_185	840 (428-488/247-307 ; 575-702/397-524)	EIN3-like protein (15425735)
			uni_CA_187	753 (434-632/96-294)	EIL2 (14280042)
uni_CA_158	766 (2-739)	expressed protein (15219020)	uni_CA_189	657 (605-713/1-109)	expressed protein (15219020)
uni_CA_159	612 (44-307)	histone H3.2 (15236103)	uni_CA_162	734 (42-441/102-501)	same
			uni_CA_160	945 (44-417/127-500)	same
			uni_CA_161	726 (71-426/132-487)	histone H3 (15232146)
			uni_CA_190	723 (43-300/71-333 ; 329-426/362-459)	histone H3 (15232146)
			uni_CA_191	667 (43-300/67-324 ; 329-426/353-450)	histone H3(15232146)
			uni_CA_192	755 (43-285/59-301 ; 329-426/345-442)	histone H3(15232146)
uni_CA_160	667 (127-390)	histone H3 (15232146)	uni_CA_190	723 (11-667/20-678)	same
			uni_CA_161	726 (52-477/89-514)	same
			uni_CA_192	755 (64-477/56-469)	same
			uni_CA_162	734 (95-450/131-486)	histone H3.2 (15236103)
			uni_CA_160	945 (227-465/286-524)	histone H3.2 (15236103)
			uni_CA_163	798 (67-450/41-424)	histone H3.2 (15236103)
			uni_CA_159	612 (67-324/43-300 ; 353-450/329-426)	histone H3.2 (15236103)
uni_CA_161	726 (105-365)	ENSANGP00000016056 histone (31210957)	uni_CA_192	755 (88-512/43-467)	histone H3 (15232146)
			uni_CA_190	723 (89-512/61-484)	histone H3 (15232146)
			uni_CA_191	667 (89-512/52-475)	histone H3 (15232146)
			uni_CA_160	945 (105-178/127-200 ; 255-502/277-524)	histone H3.2 (15236103)
			uni_CA_163	798 (132-487/69-424)	histone H3.2 (15236103)
			uni_CA_162	734 (104-487/103-486)	histone H3.2 (15236103)
			uni_CA_159	612 (132-487/71-426)	histone H3.2 (15236103)
uni_CA_162	734 (104-367)	histone H3.2 (15236103)	uni_CA_163	798 (102-501/40-439)	same
			uni_CA_159	612 (102-501/42-441)	same
			uni_CA_160	945 (104-515/127-538)	same
			uni_CA_166	1,780 (104-515/82-493)	rubisco small subunit (24940138)
			uni_CA_190	723 (131-486/104-459)	histone H3 (15232146)
			uni_CA_191	667 (131-486/95-450)	histone H3 (15232146)
			uni_CA_192	755 (103-486/59-442)	histone H3 (15232146)
			uni_CA_161	726 (103-486/104-487)	histone H3 (15232146)
			uni_CA_164	1,599 (102-171/499-568)	putative Ruv DNA-helicase (7208771)
			uni_CA_165	1,837 (102-171/454-523)	putative Ruv DNA-helicase (7208771)
uni_CA_163	798 (96-359)	histone H3.2 (15236103)	uni_CA_159	612 (1-607/3-605)	same
			uni_CA_162	734 (40-439/102-501)	same
			uni_CA_160	945 (42-415/127-500)	same
			uni_CA_161	723 (69-424/132-487)	histone H3 (15232146)
			uni_CA_191	726 (41-424/67-450)	histone H3 (15232146)
			uni_CA_192	667 (41-283/59-301 ; 327-424/345-442)	histone H3 (15232146)
uni_CA_164	1,599 (571-500)	putative Ruv DNA-helicase (7208771)	uni_CA_165	1,837 (45-573/1-528 ; 617-903/534-820 ; 952-1333/869-1250 ; 1333-1598/1374-1642)	Same
			uni_CA_193	1,356 (1333-1599/299-568 ; 1276-1333/13-70)	no hits found
			uni_CA_194	920 (607-715/944-1052 ; 712-771/1149-1208)	same
			uni_CA_195	798 (657-835/1-179 ; 1162-1599/180-620)	same
			uni_CA_196	787 (607-715/247-355 ; 712-771/452-511)	same
			uni_CA_197	777 (1333-1403/295-365)	no hits found

Table S5. Continuation.

uni_CA_165	1,837 (526-455)	putative Ruv DNA-helicase (7208771)	uni_CA_193	920 (1193-1374/13-194 ; 1373-1837/298-761)	no hits found
			uni_CA_164	1,599 (1-67/45-111 ; 120-528/165-573 ; 534-805/617-888 ; 870-1250/953-1333 ; 1374-1642/1333-1598)	same<br>same
			uni_CA_195	798 (574-752/1-179 ; 1079-1250/180-351 ; 1374-1813/351-788)	no hits found
			uni_CA_197	777 (1371-1444/292-365)	same
			uni_CA_196	787 (534-632/257-355 ; 629-688/452-511)	same
			uni_CA_194	1,356 (534-632/954-1052 ; 629-688/1149-1208)	same
uni_CA_166	1,780 (82-345)	histone H3 (296083584)	uni_CA_162	734 (82-493/104-515)	same

unigenes containing TE-cassette insertions ("query"): arbitrary identification of the unigenes identified by RepeatMasker (Lopes et al., 2008) and tBLASTx (this study), length followed by localization of the TE-cassette insertion site into unigene and results of the BLASTx searches of the unigenes against nr databank.

unigenes related those containing TE by BLASTn comparisons ("subject"): arbitrary identification of the unigene, length followed by regions of similarity between "query"/"subject" (between brackets); results of the BLASTx searches of the unigenes against nr databank (same= means unigenes with same molecular function detected for the "query"). Unigenes marked in green means putative alternative *splicing* events (Figure S6 in File S3); unigenes marked in blue means that the unigenes present one or more similarity regions, however, these regions finish before of the initial or begin after the TE insertion (Figure 1B) and, finally, unigene marked in red means *EST* cluster that also harbor TE-cassette as the *query*.

Table S6. List of unigenes containing or not TE-cassette insertions in *C. canephora*.

unigenes containing cassette-TE insertions ("query")			unigenes related those containing TE by BLASTn comparisons ("subject")		
Identification	unigene length in bp (TE insertion)	First protein hit in BLASTx searches (Genbank accession #)	Identification	unigene lenght (bp) [regions of similarity between "query"/"subject"]	First protein hit in BLASTx searches (Genbank accession #)
**unigenes identified by RepeatMasker**					
uni_CC_001	792 (171-252)	RNA Binding Protein 47 (9663769)	uni_CC_060	1,546 [16-785/88-869]	DNA binding protein ACBF-like (82621158)
			uni_CA_198	2,158 [37-160/113-236 e 214-785/290-867]	unknown protein (15226402)
uni_CC_002	811 (574-672)	no hits found	no hits found		
uni_CC_003	792 (12-99)	TTN9 (TITAN9) (22331190)	no hits found		
uni_CC_004	775 (316-425)	Ribosomal protein L21 family protein, putative 50S ribosomal protein L21 (115465385)	uni_CC_061	482 [126-402/9-276 ; 413-621/263-471]	same
uni_CC_005	815 (96-195)	Unknown (21536685)	no hits found		
uni_CC_006	707 (102-149)	adenylyl cyclase-like (57899387)	no hits found		
uni_CC_007	1,126 (492-567)	Cwf15/Cwc15 cell cycle control protein (92872088)	uni_CC_062	987 [114-212/8-106 ; 319-930/106-720]	same
			uni_CC_063	908 [319-520/293-494 ; 114-203/195-284]	same
			uni_CA_199	1087 [1-60/54-113 e 111-791/285-961]	unknown protein (18379014)
uni_CC_008	807 (58-103)	Unknown (18379014)	no hits found		
uni_CC_009	631 (363-408)	Unknown (21554182)	uni_CC_019	486 [103-583/12-486]	hypothetical protein MA4_112I10.14 (102140015)
uni_CC_010	867 (479-566)	major latex protein homolog (3064039)	no hits found		
uni_CC_011	1,045 (59-105)	no hits found	no hits found		
uni_CC_012	102 (6-79)	no hits found	no hits found		
uni_CC_013	429 (271-427)	no hits found	no hits found		
uni_CC_014	487 (364-405)	no hits found	no hits found		
uni_CC_015	477 (383-476)	no hits found	no hits found		
uni_CC_016	506 (32-243)	no hits found	no hits found		
uni_CC_017	467 (28-85)	no hits found	no hits found		
uni_CC_018	486 (132-226)	no hits found	uni_CC_064	472(11-129/391-271)	no hits found
			uni_CC_045	995(115-174/158-217)	Capip2 (47558819)
			uni_CC_048	1,034 (116-174314-256)	13-lipoxygenase (1495804)
uni_CC_019	486 (272-317)	hypothetical protein MA4_112I10.14 (102140015)	uni_CC_009	631 [12-486/103-583]	unknown (21554182)
uni_CC_020	200 (8-58)	hypothetical protein (115640798)	uni_CC_065	200 [1-200/1-200]	same
uni_CC_021	511 (31-114)	no hits found	no hits found		
uni_CC_022	707 (132-168)	no hits found	uni_CC_066	526 [264-611/109-452]	no hits found
uni_CC_023	777 (521-608)	no hits found	uni_CC_067	669 [9-152/16-159 ; 638-757/159-278]	no hits found
uni_CC_024	842 (271-335)	no hits found	uni_CC_068	723 [285-627/11-354]	no hits found
			uni_CC_069	874 [258-426/71-239]	no hits found
uni_CC_025	883 (580-733)	14-3-3 homologues mediates signal transduction by binding to phosphoserine-containing proteins (92891732)	No hits found		

Table S6. Continuation.

uni_CC_026	886 (17-69)	no hits found	No hits found		
uni_CC_027	793 (676-770)	structural constituent of ribosome (18401997)	No hits found		
uni_CC_028	331 (14-102)	no hits found	No hits found		
uni_CC_029	529 (46-161)	no hits found	uni_CC_070	480 [326-515/480-291]	same
			uni_CC_071	968 [248-524/655-380]	same
			uni_CC_072	701 [107-231/576-451 ; 334-524/441-252]	same
			uni_CC_073	457 [107-231/304-428]	same
			uni_CC_074	579 [107-231/244-368]	same
			uni_CC_075	897 [163-225/8-70 ; 248-414/76-243]	same
			uni_CC_076	502 [144-231/390-477]	same
			uni_CC_077	518 [278-386/275-165]	same
uni_CC_030	441 (10-221)	no hits found	No hits found		
uni_CC_031	469 (60-112)	no hits found	No hits found		
uni_CC_032	886 (628-764)	unknown protein (14334448)	No hits found		
uni_CC_033	341 (211-300)	putative proline-rich protein APG isolog (10638955)	No hits found		
uni_CC_034	1,086 (162-218)	no hits found	No hits found		
uni_CC_035	1,075 (482-539)	unknown protein (116830383)	No hits found		
uni_CC_036	1,034 (10-316)	no hits found	No hits found		
uni_CC_037	1,056 (876-926)	protein binding / transporter, Protein transport protein Sec24-like , putative Sec24-like COPII protein (30680129)	uni_CC_078	1,152 [33-715/57-741]	same
			uni_CC_079	919 [529-719/534-725]	same
uni_CC_038	1,247 (105-174)	26S proteasome regulatory complex component, contains PCI domain (90265119)	uni_CC_080	508 [52-127/20-95; 266-678/96-508]	26S proteasome regulatory particle non-ATPase subunit7 (17297977)
uni_CC_039	827 (446-497)	hexokinase 6 (45387415)	No hits found		
uni_CC_040	830 (347-471)	putative CEO protein (alternative splicing products) (29126336)	uni_CC_081	1,290 [561-771/1072-1289]	unknown protein (158110143)
			uni_CA_200	833 [561-830/298-567]	CEO protein (29126336)
			uni_CA_201	1,725 [12-346/1017-1351 e 468-686/1507-1725]	no hits found
			uni_CA_202	2,737 [561-830/2082-2352]	CEO protein (11044957)
uni_CC_041	589 (257-332)	CID11; RNA binding (79319100)	No hits found		
uni_CC_042	655 (311-632)	echinoderm microtubule associated protein like 5 (68355594)	No hits found		
uni_CC_043	367 (47-107)	ribosomal protein L7 (445613)	uni_CC_082	1,028 [16-69/12-65; 75-227/43-198]	60S ribosomal protein L7 (77999275)
uni_CC_044	944 (820-898)	DNA binding, phospatase 2A inhibitor (15221298)	No hits found		
uni_CC_045	995 (175-250)	Capip2 (47558819)	uni_CC_083	854 [9-204/655-850]	no hits found
			uni_CC_048	1,034 [11-251/459-222]	13-lipoxygenase (1495804)
			uni_CC_084	523 [10-172/299-459]	no hits found
			uni_CC_085	297 [10-172/97-259]	no hits found
			uni_CC_018	486 [158-217/115-174]	no hits found

Table S6. Continuation.

uni_CC_046	1,012 (42-78)	no hits found	uni_CC_086	487 [830-1012/136-328]	no hits found
			uni_CC_087	298 [892-1012/14-136]	no hits found
			uni_CC_088	879 088 [836-1007/586-761]	no hits found
			uni_CC_089	469 [610-829/285-63]	no hits found
			uni_CC_090	527 [727-829/103-205]	no hits found
			uni_CC_091	434 [830-995/105-275]	no hits found
			uni_CC_092	480 [830-987/210-371]	no hits found
uni_CC_047	1,413 (567-681)	cell wall-plasma membrane linker protein (11994733)	No hits found		
			uni_CA_203	1,537 [9-437/160-588 e 414-645/598-829 e 755-1280/906-1429]	same
uni_CC_048	1,034 (165-294)	13-lipoxygenase (1495804)	uni_CC_093	958 [614-1034/252-673]	Lipoxygenase (32454714)
			uni_CC_094	1,140 [614-988/402-777]	Lipoxygenase (14589309)
			uni_CC_083	854 [269-614/850-501]	no hits found
			uni_CC_095	893 [298-605/54-363]	no hits found
			uni_CC_096	685 [347-611/525-259]	no hits found
			uni_CC_045	995 [222-469/251-11]	Capip2 (47558819)
			uni_CC_097	221 [415-614/221-21]	no hits found
			uni_CC_084	523 [319-596/441-162]	no hits found
			uni_CC_085	297 [319-538/240-19]	no hits found
			uni_CC_098	342 [9-149/165-25]	Retrosat (28558781)
			uni_CC_099	751 [13-226/229-16]	Retrosat (28558781)
			uni_CC_100	475 [411-520/118-9]	no hits found
			uni_CC_101	609 [410-538/263-135]	membrane-associated salt-inducible (3249103)
			uni_CC_102	562 [397-538/545-404]	no hits found
			uni_CC_018	486 [256-314/174-116]	no hits found
uni_CC_049	716 (117-210)	Nascent polypeptide-associated alpha subunit like protein complex NAC; UBA-like (87241023)	uni_CC_103	804 [8-716/12-722]	same
			uni_CA_204	852 [6-118/1-113 e 158-716/147-710]	nascent polypeptide associated complex alpha chain (34907258)
			uni_CA_205	903 [6-118/28-140 e 159-716/178-740]	nascent polypeptide associated complex alpha chain (34907258)
uni_CC_050	500 (394-477)	No hits found	uni_CC_104	575 [352-500/9-157]	no hits found
uni_CC_051	504 (205-287)	Proteína unknown (18417290)	uni_CC_105	593 [85-375/146-436]	same
			uni_CC_106	475 [389-174/391-176]	no hits found
uni_CC_052	575 (51-134)	No hits found	uni_CC_050	500 [155-9/498-352]	no hits found
uni_CC_053	910 (79-218)	unknown protein (4734014)	No hits found		
uni_CC_054	1,049 (460-512)	ARRZ-1A; RNA binding / nucleotide binding, RNA-binding protein (15231557)	No hits found		
uni_CC_055	967 (536-626)	No hits found	No hits found		
uni_CC_056	368 (154-214)	Yippee-like protein (77555722)	No hits found		
uni_CC_057	1,966 (957-1009)	hydrolase-like protein (53828585)	No hits found		
uni_CC_058	1,129 (161-284)	putative SCO1 protein (18398306)	No hits found		
uni_CC_059	1,169 (440-524)	No hits found	No hits found		

unigenes containing TE-cassette insertions (“query”): arbitrary identification of the unigenes identified by RepeatMasker (Lopes et al., 2008) and tBLASTx (this study), length followed by localization of the TE-cassette insertion site into unigene and results of the BLASTx searches of the unigenes against nr databank.

unigenes related those containing TE by BLASTn comparisons (“subject”): arbitrary identification of the unigene, length followed by regions of similarity between “query”/”subject” (between brackets); results of the BLASTx searches of the unigenes against nr databank (same= means unigenes with same molecular function detected for the “query”). Unigenes marked in green means putative alternative *splicing* events (Figure S6 in File S3); unigenes marked in blue means that the unigenes present one or more similarity regions, however, these regions finish before of the initial or begin after the TE insertion (Figure 1B) and, finally, unigene marked in red means *EST* cluster that also harbor TE-cassette as the *query*.
